# LC-MS guided isolation of three pairs of enantiomeric alkaloids from *Macleaya cordata* and their enantioseparations, antiproliferative activity, apoptosis-inducing property

**DOI:** 10.1038/s41598-017-15423-4

**Published:** 2017-11-13

**Authors:** Chunmei Sai, Dahong Li, Shengge Li, Tong Han, Yongzhi Guo, Zhanlin Li, Huiming Hua

**Affiliations:** 1 School of Pharmacy, Jining Medical University, Rizhao, 276826 Shandong Province People’s Republic of China; 2 Key Laboratory of Structure-Based Drug Design and Discovery, Ministry of Education, Shenyang Pharmaceutical University, Shenyang, 110016 Liaoning Province People’s Republic of China

## Abstract

(±)-Macleayins F–H (**1**–**3**), three pairs of new enantiomeric alkaloid dimers, along with four known alkaloids (**4**–**7**) as their plausible biogenetic precursors, were isolated from the aerial parts of *Macleaya cordata*. Compounds **1**–**3** were obtained under the guidance of LC-MS investigation, and their structures were elucidated by analysis of the 1D and 2D NMR spectroscopic data. The racemic mixtures were successfully separated by chiral HPLC, and the absolute configurations of enantiomers were determined by electronic circular dichroism (ECD) spectroscopy. Compounds **1**–**7** showed antiproliferative activity against HL-60 with IC_50_ values of 1.34–41.30 *μ*M, especially compounds **1**–**2** exhibited the best inhibitory activity against HL-60 cell lines. In addition, the preliminary mechanism investigation for compound **2** using Annexin V/7-AAD double-staining assay, DAPI staining assay and JC-1 staining method, indicated that **2** inhibited cancer cell proliferation potentially through inducing apoptosis *via* the mitochondria-related pathway and arrested cell cycle of HL-60 cells at S phase.

## Introduction

**Figure 1:**
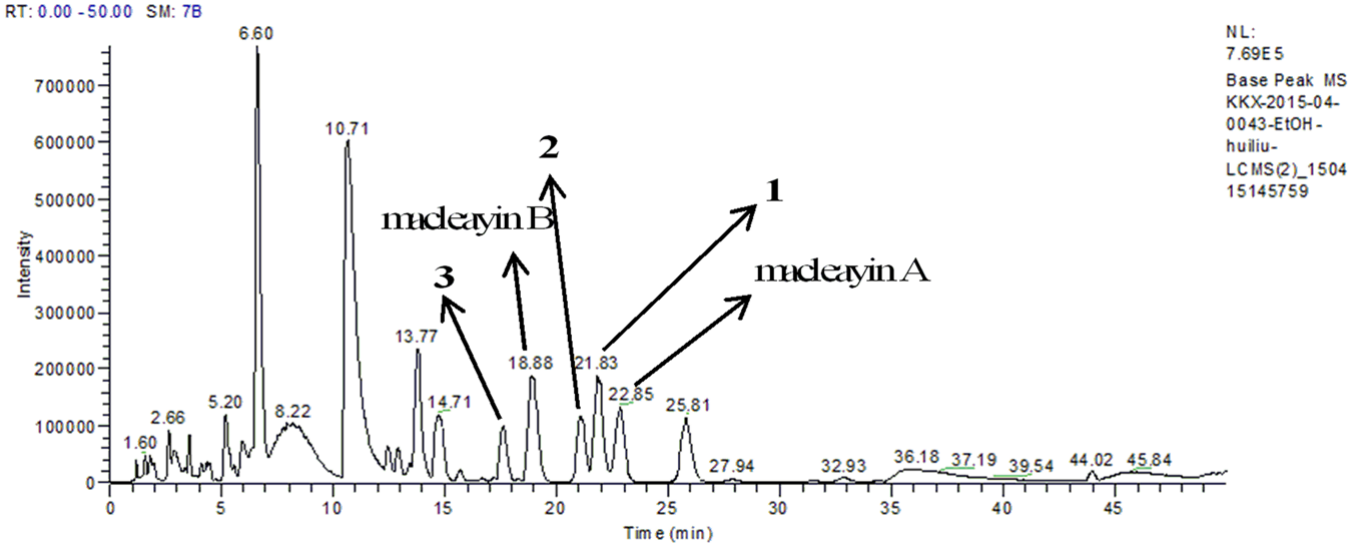
LC-MS analysis chromatogram of the crude ethanol extract from *M*. *cordata*.

**Figure 2:**
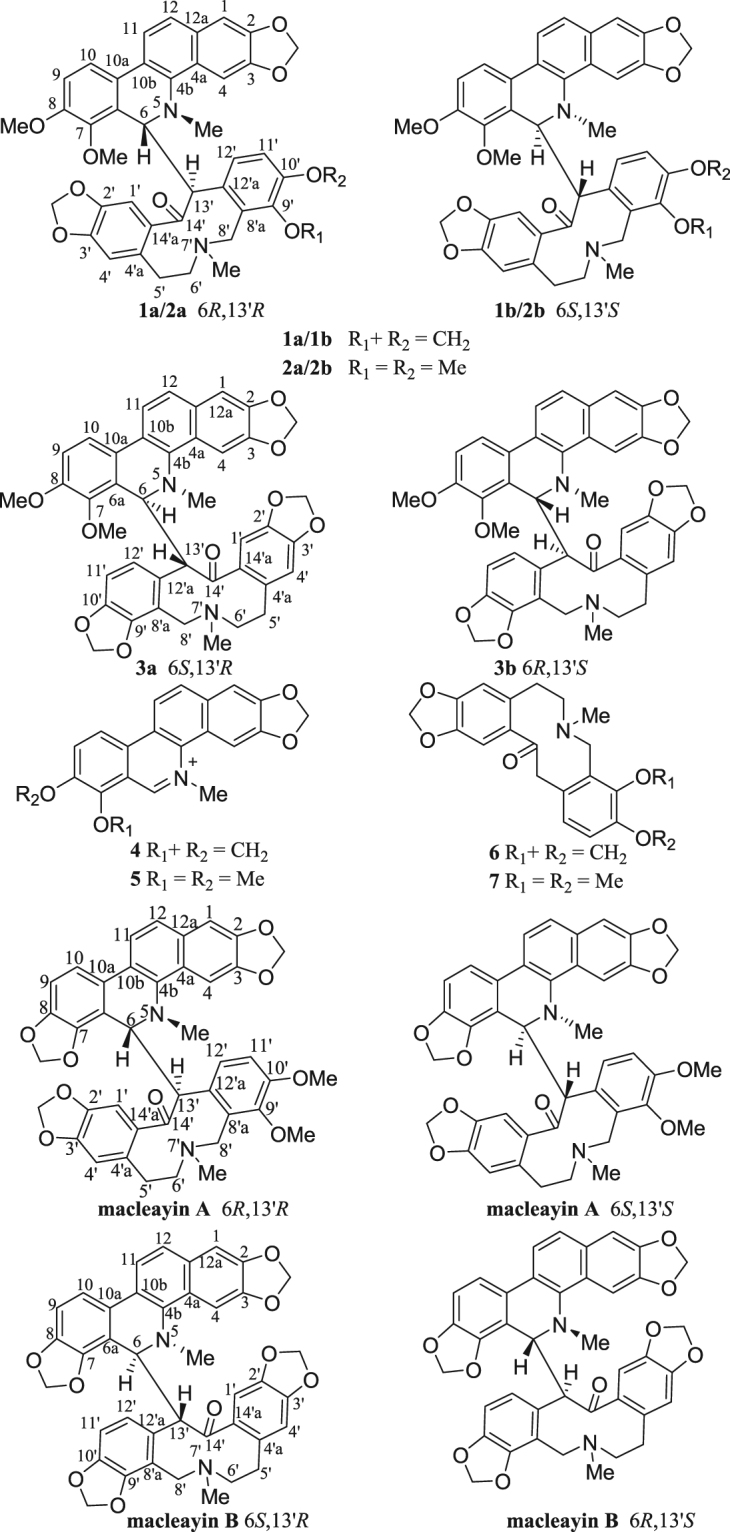
Structures of compounds **1**–**7** and macleayins A, B.

Benzophenanthridine and protopine alkaloids occur in *Macleaya cordata* (Willd) R. Br. and have been investigated for their intriguing bioactivity, such as anticancer, anti-bacterial, insecticidal, and anti-inflammatory effects^1,2^. *Macleaya cordata* is a perennial plant in the family of Papaveraceae, which has been used in folk medicine for the treatment of cervical cancer, thyroid cancer, inflamed wounds, ringworm infection, arthritis, and trichomonas vaginalis^3,4^. In our previous investigation, two pairs of enantiomeric alkaloid dimers (macleayins A and B) with cytotoxicity were isolated from *M*. *cordata* ^5^. In our present study, LC-MS- guided fractionation (Fig. 1) led to the isolation of three pairs of analogous dimers, macleayins F–H (**1**–**3**) biogenetically derived from a benzophenanthridine and protopine alkaloid through C6–C_13′_ linkage. Four possible biogenetic precursors, sanguinarine, chelerythrine, protopine, and allocryptopine (**4**–**7**) were obtained (Fig. 2). Herein, we reported the isolation, and structural elucidation of new compounds. In addition, the antiproliferative properties and action mechanism were also investigated.

## Results and Discussion

### Structural Elucidation of Compounds 1a/1b–3a/3b

Macleayin F (**1**) was isolated as white amorphous powder, with the molecular formula of C_41_H_36_N_2_O_9_, deduced from HRESIMS [M + H]^+^ (*m/z* 701.2491, calcd for 701.2494), appropriate for 25 degrees of unsaturation. The IR spectrum indicated the presence of ketone carbonyl (1656 cm^−1^), methylenedioxyl group (2792, 941 cm^−1^), and aromatic ring (1616, 1486, 1462 cm^−1^). In the UV spectrum, the absorption maxima at 227 and 287 nm were detected. The HRESIMS/MS spectrum exhibited the fragment peak at *m/z* 348.1281 assigned to chelerythrine^6^. The ^1^H NMR (Table 1) spectrum revealed the presence of three AB spin systems of aromatic protons in *ortho*-position ((*δ*_H_ 6.68 (br d, *J* = 7.3 Hz, H-9) and 7.23 (br d, *J* = 7.3 Hz, H-10), 7.46 (d, *J* = 8.5 Hz, H-12) and 7.61 (d, *J* = 8.5 Hz, H-11), 6.97 (br d, *J* = 8.0 Hz, H-11′) and 7.50 (br s, H-12′)), four aromatic protons in singlet (*δ*_H_ 7.05, 6.85, 6.83, and 6.21), two sp^3^ methine protons (*δ*_H_ 5.13 (d, *J* = 9.0 Hz) and 4.59 (d, *J* = 9.0 Hz)), as well as three methylenedioxyl groups ((*δ*_H_ 5.99 (d, *J* = 1.1 Hz), 5.96 (d, *J* = 1.1 Hz), 5.91 (d, *J* = 1.1 Hz), 5.83 (d, *J* = 1.1 Hz), 5.87 (d, *J* = 1.4 Hz), and 5.82 (d, *J* = 1.4 Hz)), two methoxyl groups (*δ*_H_ 3.94 and 3.83), and two *N*-methyl groups (*δ*_H_ 2.51 and 1.63). The ^13^C NMR (Table 1) combined with the HSQC spectroscopic data identified twenty-eight aromatic, three methylenedioxyl, two methoxyl, two *N*-methyl, three methylene, and two sp^3^ methine carbons (Table 1). Considered its molecular formula, one carbonyl carbon signal was absent in its ^13^C NMR spectrum. The above data suggested that **1** was a dimeric alkaloid consisting of a chelerythrine and a protopine moiety^5^.

**Table 1:** ^1^H [600 MHz, *δ* in ppm, Mult. (*J* in Hz)] and ^13^C NMR (150 MHz, *δ* in ppm) data of **1**–**3** in CDCl_3_. ^−a^No signal observed in ^1^H NMR and ^13^C NMR spectra.

No.	1		2		3	
	*δ*_H_ (mult., *J*, in Hz)	*δ*_C_	*δ*_H_ (mult., *J*, in Hz)	*δ*_C_	*δ*_H_ (multi, *J*, in Hz)	*δ*_C_
1	7.05 (s)	104.2	7.03 (s)	104.1	7.01 (s)	104.0
2		147.5		147.4		147.2
3		147.9		148.0		147.4
4	6.85 (s)	100.8	6.77 (s)	100.8	5.29 (br s)	100.5
4a		127.6		127.8		127.1
4b		139.4		139.4		139.5
6	5.13 (d, 9.0)	58.0	5.14 (d, 10.3)	57.8	^−a^	63.8
6a		126.7		127.0		125.6
7		146.3		146.3		146.8
8		152.1		152.0		152.1
9	6.68 (br d, 7.3)	111.7	6.66 (br s)	111.5	6.84 (d, 8.4)	112.6
10	7.23 (br d, 7.3)	116.6	7.23 (br s)	118.5	7.39 (d, 8.4)	118.9
10a		125.0		125.0		125.4
10b		124.3		124.5		124.0
11	7.61 (d, 8.5)	119.8	7.63 (d, 8.5)	119.9	7.78 (d, 8.4)	119.8
12	7.46 (d, 8.5)	123.8	7.45 (d, 8.5)	123.7	7.48 (d, 8.4)	124.2
12a		131.0		130.8		130.9
1′	6.83 (s)	111.7	6.92 (s)	112.1	^−a^	^−a^
2′		145.6		145.4		^−a^
3′		148.3		147.8		^−a^
4′	6.21 (s)	109.8	6.19 (s)	109.7	5.89 (s)	110.8
4′a		133.7		134.1		^−a^
5′	2.37–2.31 (m) 1.93 (br s)	33.4	2.50–2.33 (m) 1.86–1.65 (m)	33.7	2.23–1.59 (m)	^−a^
6′	2.37–2.31 (m) 1.85 (br s)	56.9	2.50–2.33 (m) 1.86–1.65 (m)	57.2	2.23–1.59 (m)	^−a^
8′	3.07 (d, 13.2) 2.32 (br s)	48.5	3.09 (d, 13.3) 2.50–2.33 (m)	48.3	3.21 (br s) 3.02 (br s)	^−a^
8′a		118.5		130.6		^−a^
9′		145.0		147.0		^−a^
10′		146.0		150.5		150.1
11′	6.97 (br d, 8.0)	107.1	7.07 (d, 8.3)	110.8	6.81 (d, 6.6)	107.7
12′	7.50 (br s)	122.5	7.61 (d, 8.3)	125.2	7.83 (br s)	^−a^
12′a		132.9		132.1		^−a^
13′	4.59 (d, 9.0)	52.8	4.47(d, 10.3)	52.9	5.01 (br s)	^−a^
14′		^−a^		^−a^		^−a^
14′a		135.5		135.6		^−a^
5-*N*-CH_3_	2.51 (s)	41.1	2.50 (s)	40.8	2.56 (s)	43.1
7′-*N*-CH_3_	1.63 (s)	41.7	1.49 (s)	41.5	1.89 (s)	41.4
2,3-OCH_2_O-	5.99 (d, 1.1) 5.96 (d, 1.1)	101.1	5.93–5.81 (m)	101.0	5.99–5.76 (m)	101.1
7-OCH_3_	3.94 (s)	61.0	3.93 (s)	61.8	3.77 (s)	61.1
8-OCH_3_	3.83 (s)	55.9	3.81 (s)	55.7	3.65 (s)	56.1
2′,3′-OCH_2_O-	5.91 (d, 1.1) 5.83 (d, 1.1)	101.1	5.93–5.81 (m)	100.9	5.99–5.76 (m)	101.6
9′,10′-OCH_2_O- or –OCH_3_	5.87 (d, 1.4) 5.82 (d, 1.4)	100.6	3.50 (s) 3.95 (s)	60.9 56.0	5.99–5.76 (m)	100.9

**Figure 3:**
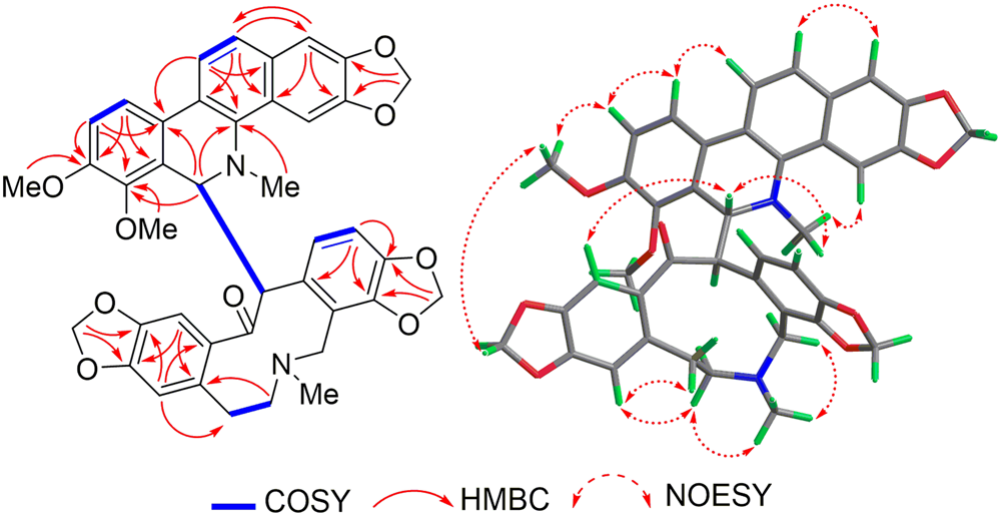
HMBC,^1^H-^1^H COSY, and NOESY correlations of macleayin F (**1**).

The HMBC correlations (Fig. 3) of H-1/C-3, C-4a, C-12; H-10/C-6a, C-8; H-11/C-4b, C-10a, C-12a; H-12/C-1, C-4a, C-10b; 7-OCH_3_/C-7; 8-OCH_3_/C-8; -*N*CH_3_/C-4b, and from the protons of OCH_2_O (*δ*_H_ 5.99 and 5.96) to C-2, and C-3, as well as the^1^H−^1^H COSY correlations (Fig. 3) of H-9/H-10 and H-11/H-12, evidenced the presence of chelerythrine moiety. This was furthermore confirmed by the fragment peak at *m/z* 348.1281 in the HRESIMS/MS (Supplementary Figure S13). In the ^1^H NMR spectra of **1**, the signals in the high field region were substantially overlapped. Hence, the presence of protopine structure could not be completely assigned with the observed HMBC cross-peaks of H-1′/C-3′, C-4′a; H-4′/C-2′, C-5′, C-14′a; H-11′/C-9′, C-10′, C-12′a; as well as cross-peaks of two methylenedioxys at *δ*_H_ 5.91, 5.83/C-2′, C-3′ and *δ*_H_ 5.87, 5.82/C-9′, C-10′. Comparison of the NMR and HRESIMS data of **1** with those of macleayins A and B^5^ which were previously obtained as a single crystal molecule from *M*. *cordata* indicated that **1** and macleayin B possessed protopine moiety. In addition, the^1^H-^1^H COSY of H-6/H-13′ proved the direct linkage of C6–C13′. Similarly, one carbonyl and several crucial HMBC correlations were not observed in **1**. Accordingly, the planar structure of compound 1 was defined as shown.

**Figure 4:**
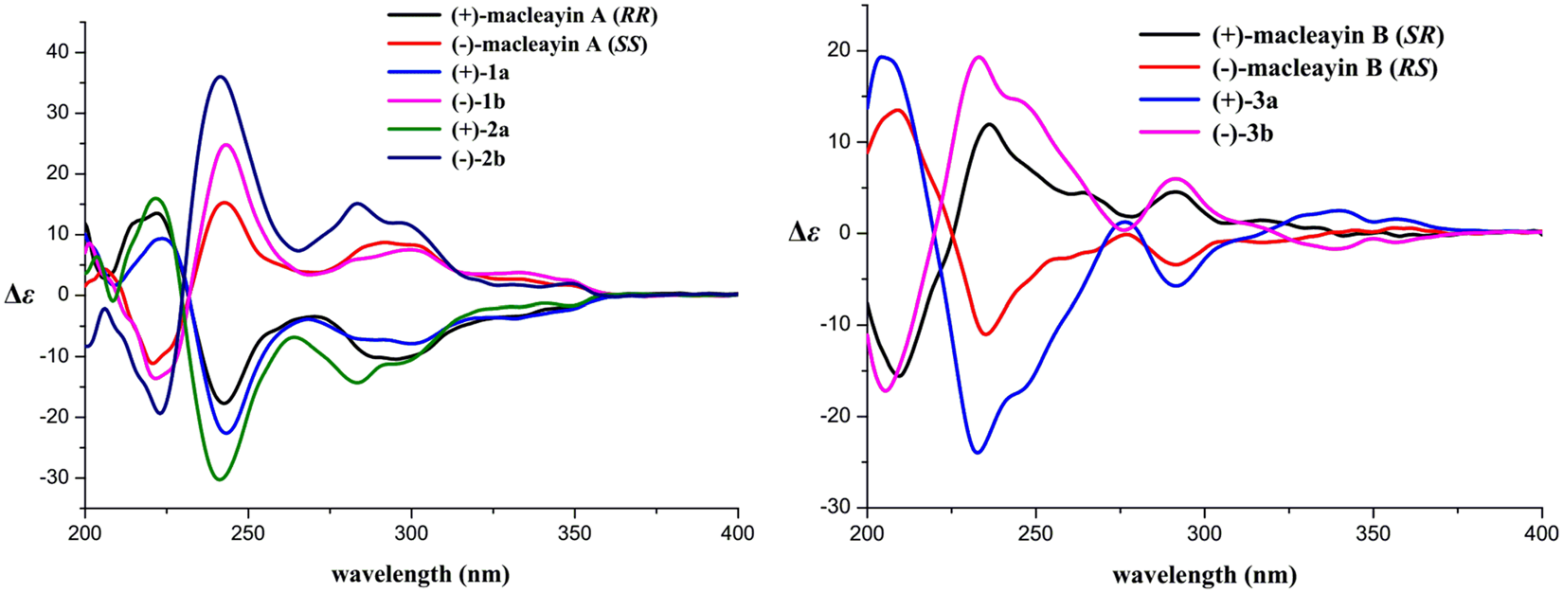
CD spectra for compounds **1a**, **1b**, **2a**, **2b**, **3a**, **3b**, and (+)-/(−)-macleayin A, (+)-/(−)-macleayin B.

The relative stereochemistry of **1** was established by the NOESY experiment (Fig. 3). The coupling constant (*J* = 9.0 Hz) and no NOE correlation between H-6 and H-13′, suggested that H-6 and H-13′ may be on the opposite side. The proposed relative stereochemistry is a reasonable hypothesis, as well as the fact that this is due to the lacunar nature of the available data set. Compound **1** was a racemic mixture like macleayins A and B due to the lack of optical rotation and Cotton effect (CE)^7^. Subsequently, the enantioseparation of **1** was performed on a chiral column (Daicel Chiralpak IB) to yield the enantiomers **1a** and **1b** in an approximate 1:1 ratio (Supplementary Figure S4)^8^, which were opposite in terms of optical rotation (**1a**:${[\alpha ]}_{{\rm{D}}}^{{\rm{20}}}$ + 274 (*c* 0.07, MeOH), **1b**: ${[\alpha ]}_{{\rm{D}}}^{{\rm{20}}}$ −248 (*c* 0.07, MeOH)). The assignments of their absolute configuration at the stereogenic centers were determined by comparing the ECD spectra (Fig. 4) of both enantiomers with those of two similar compounds (+)-macleayin A and (−)-macleayin A. The measured ECD curves of **1a** and **1b** matched with that of (6 *R*,13′*R*)-macleayin A and (6 *S*,13′*S*)-macleayin A, respectively. Hence, the absolute configuration for 1a (6 *R*,13′*R*) and **1b** (6 *S*,13′*S*) were unambiguously determined as shown in Fig. 1 and named (+)-macleayin F and (−)-macleayin F, respectively.

Macleayin G (**2**) was obtained as white amorphous powder. The molecular formula of **2** was established as C_42_H_40_N_2_O_9_ on the basis of positive HRESIMS at *m/z* 717.2799 [M + H]^+^ (calcd 717.2807), indicating 24 indices of hydrogen deficiency. The IR spectrum showed absorption bands due to ketone carbonyl (1652 cm^−1^), methylenedioxyl group (2794, 938 cm^−1^), and aromatic ring (1619, 1491, 1458 cm^−1^) functionalities. The UV spectrum exhibited maximum absorption at 229 and 286 nm. Its^1^H and ^13^C NMR data (Table 1) revealed structural similarity to **1**, expected that one methylenedioxy in **1** was replaced by two methoxyl groups (*δ*_H_ 3.95, 3.50; *δ*_C_ 56.0, 60.9). It was confirmed by the HMBC correlations of 8-OCH_3_ with C-8, 7-OCH_3_/H-6 with C-7; 9′-OCH_3_/H-8′/H-11′ with C-9′, and of 10′-OCH_3_/H-11′ with C-10′ (Supplementary Figure S2). Furthermore, the HRESIMS/MS showed the fragment ion peak at *m/z* 348.1306 which was recognized as chelerythrine, suggested that **2** was made up of chelerythrine and allocryptopine, which was supported by comparison of NMR data of **2** with those of macleayin B^5^. ^1^H-^1^H COSY correlations (Supplementary Figure S2) of H-6/H-13′ confirmed that two parts were connected by C-6 with C-13′. Similarly, compound **2** was proposed to be a racemic mixture on account of the lack of optical rotation and CE. Subsequently, **2a** and **2b** were obtained by chiral-phase HPLC separation in a ratio of 1:1 (Supplementary Figure S5), which exhibited the mirror image-like ECD curves and owned the opposite specific optical rotations (**2a**: ${[\alpha ]}_{{\rm{D}}}^{{\rm{20}}}$ + 238 (*c* 0.10 MeOH)), **2b**: ${[\alpha ]}_{{\rm{D}}}^{{\rm{20}}}$ **−**210 (*c* 0.10 MeOH)). Thus, the absolute configuration of **2a** and **2b** were assigned as (6 *R*,13′*R*) and (6 *S*,13′*S*) by comparing their experimental ECD spectra with those of (+)-macleayin A and (**−**)-macleayin A (Fig. 4), and given the names (+)-macleayin G and (**−**)-macleayin G, respectively.

Macleayin H (**3**) was isolated as white amorphous powder and yielded a quasi-molecular ion peak at *m/z* 701.2485 [M + H]^+^ (calcd for 701.2494) in the HRESIMS, indicative of molecular formula C_41_H_36_N_2_O_9_, the same as that of **1**. The identical fragment peak at *m/z* 348.1323 in HRESIMS/MS as **1**, together with 1D and 2D NMR data (Table 1) deduced that **3** was a similar dimer comprising chelerythrine and protopine as **1**. But the chemical shifts of H-4 and H-4′ in compound **3** were significantly shifted upfield (ca. −1.6 and −0.3 ppm), and H-13′ was shifted downfield (ca. +0.4 ppm). Compound **3** showed more similar NMR data (Supplementary Tables S1 and S2) to macleayin B than **1**, deducing that the relative configuration of **3** was the same as macleayin B. Subsequent chiral HPLC resolution (Supplementary Figure S6) of **3** afforded **3a** (${[\alpha ]}_{{\rm{D}}}^{{\rm{20}}}$ + 71 (*c* 0.11 MeOH)) and **3b** (${[\alpha ]}_{{\rm{D}}}^{{\rm{20}}}$ −94 (*c* 0.11 MeOH)). The absolute configuration at the stereocenters of **3a** (6 *S*,13′*R*) and **3b** (6 *R*,13′*S*) were established by comparison of their experimental ECD spectra with those of (+)-macleayin B and (−)-macleayin A (Fig. 4), and named (+)-macleayin H, and (−)-macleayin H, respectively.

### Antiproliferative activity

Our previous study revealed that alkaloids from *M*. *cordata* exhibited promising antiproliferative effects, so all the isolated compounds were tested for the growth inhibitory activities against HL-60, A-549, MCF-7 human cancer cell lines by the trypan blue method and MTT method^9,10^. The results were summarized in Table 2. All compounds inhibited the growth against all the tested cancer cells with IC_50_ values ranged from 1.34 to 41.30 *μ*M. In particular, **1a**, **1b**, **2a**, and **2b** showed more potent antiproliferative property against HL-60 cell lines than their biogenetic precursors **5–7**, and the enantiomers displayed similar inhibitory effects. Therefore, compound **2** was selected for further investigation on the antiproliferative mechanism in HL-60 cells.

**Table 2:** The antiproliferative activity of compounds** 1**–**7**. IC_50_ is the concentration that inhibited 50% of cell growth. Results are expressed as the mean ± SD of three independent experiments.

Comp.	IC_50_ (*μ*M)		
	HL-60	A-549	MCF-7
1a	1.70 ± 0.89	10.98 ± 1.93	25.78 ± 0.88
1b	1.34 ± 1.02		
2a	3.35 ± 0.13	9.87 ± 1.08	12.29 ± 1.06
2b	2.47 ± 0.56		
3a	6.49 ± 1.24	41.30 ± 0.29	18.88 ± 0.99
3b	8.37 ± 0.26		
4	7.71 ± 0.78	8.49 ± 0.63	5.51 ± 1.42
5	9.61 ± 2.45	9.38 ± 0.91	7.74 ± 2.75
6	8.94 ± 0.37	27.37 ± 1.05	25.19 ± 3.09
7	7.18 ± 1.03	26.06 ± 0.58	28.24 ± 1.64
5-Fu	2.8 ± 2.03	1.6 ± 1.22	17.01 ± 0.95

### Influence of compound 2 on the HL-60 cell cycle

**Figure 5:**
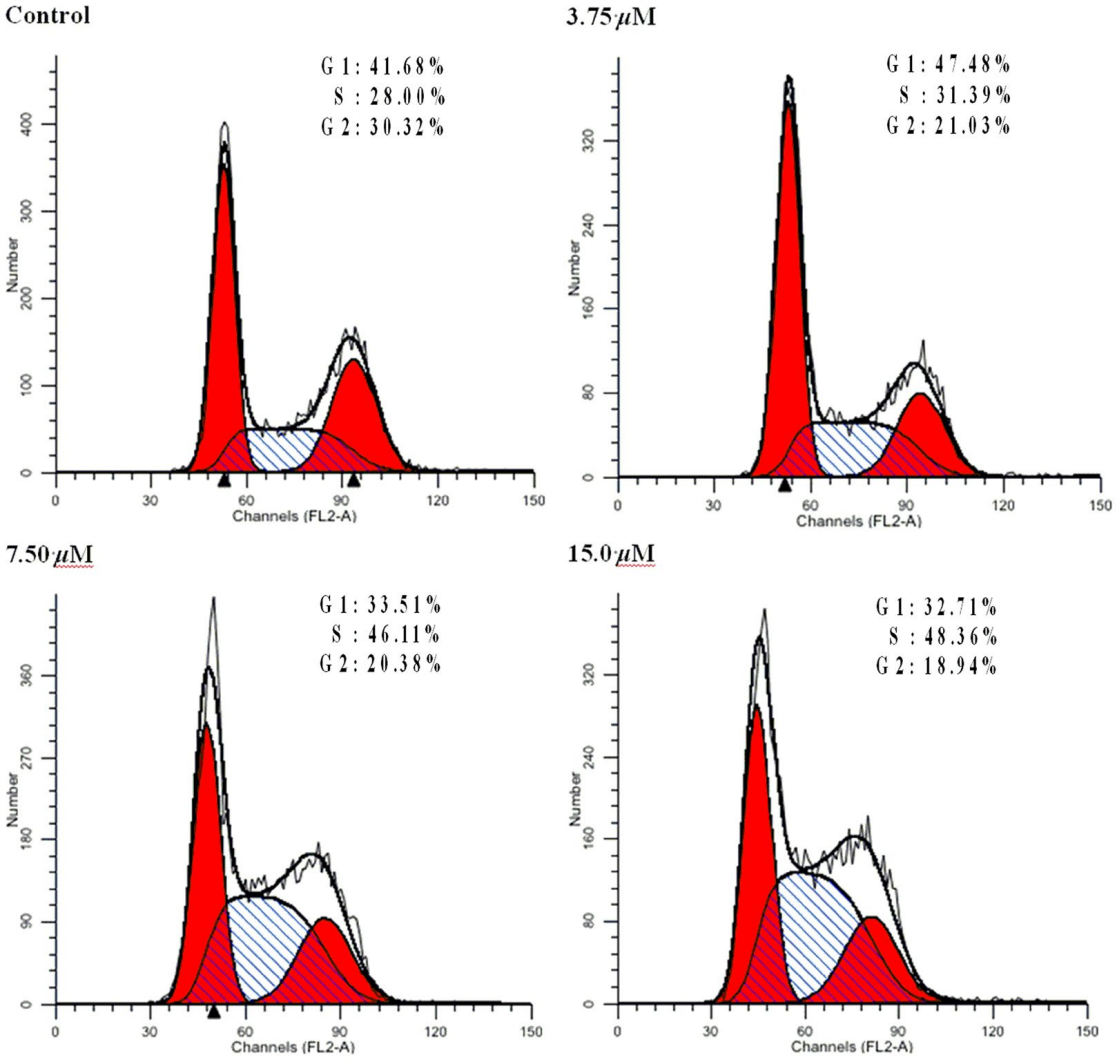
The influence of HL-60 cell cycle by compound **2**. Left red part: cell in G1 phase; Right red part: cell in G2 phase; Oblique line part: cell in S phase; White part: total cells.

Cell cycle arrest was an important sign for inhibition of proliferation and the series of events that took place in a cell leading to its division and duplication^11,12^. In order to explore whether the growth inhibition induced by **2** was caused by the regulation of the HL-60 cell cycle, the cell cycle distribution in the presence of **2** was detected by flow cytometry (Fig. 5). HL-60 cells were treated with compound **2** at concentrations of 3.75, 7.5, and 15.0 *μ*M for 72 h, which resulted in a remarkable increase of 31.49%, 46.11%, and 48.36% of cells at S phase compared with that of the control (28.00%), while there was a concomitant decline in the number of cells in G1 and G2 phases. This information indicated that compound **2** influenced cell cycle of HL-60 arrested at S phase in a dose-dependent manner.

### Induction of apoptosis by compound 2

**Figure 6:**
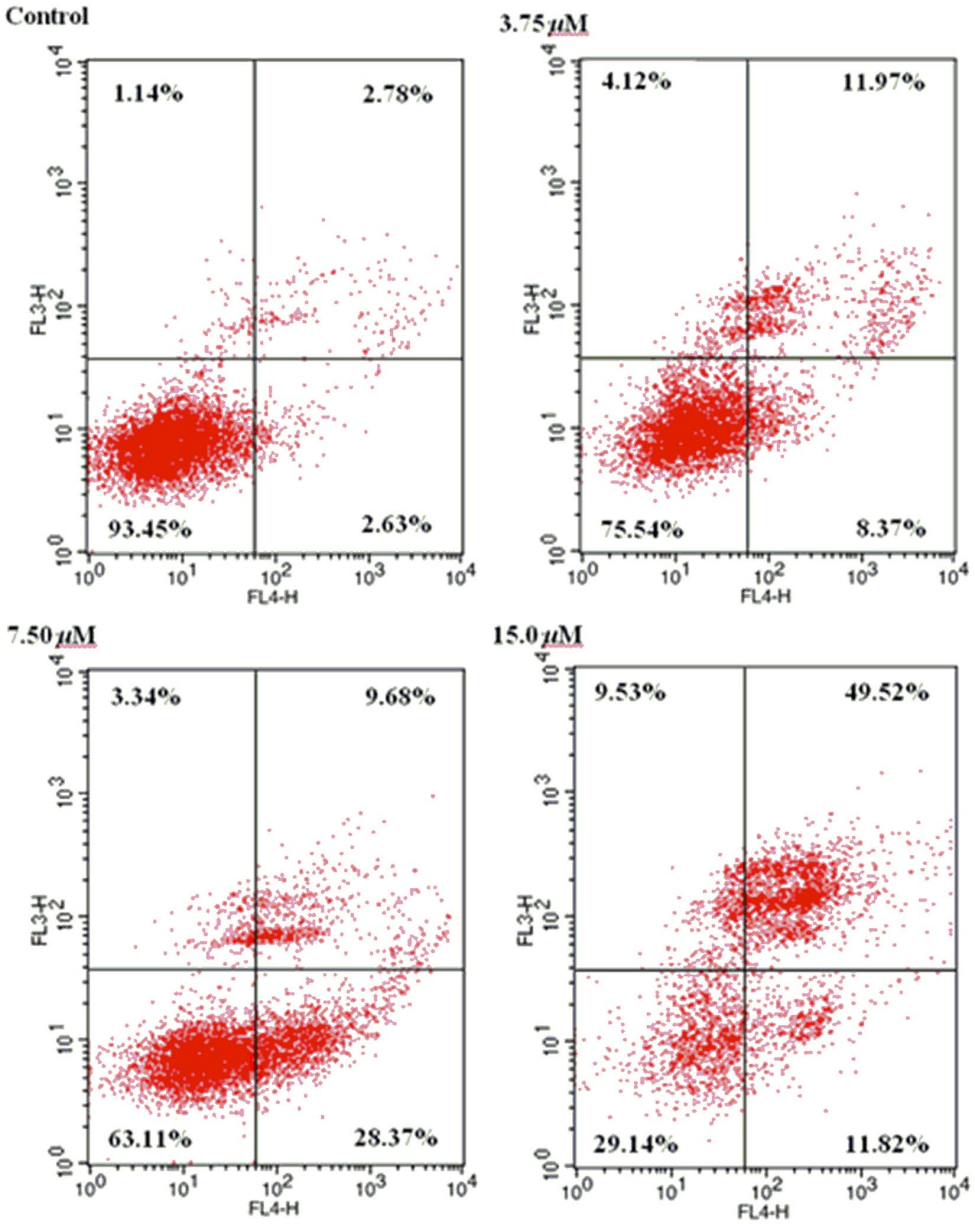
Induction of apoptosis by** 2** in HL-60 cells.

Apoptosis is an ordered and orchestrated cellular process that takes play in physiological and pathological conditions^13,14^. Therefore, drug-induced apoptosis in tumor cells is important for cancer treatment. The effects of **2** on the apoptosis of HL-60 cells by staining them with Annexin-V APC/7-AAD and analysis by flow cytometry were examined. As shown in Fig. 6, the treatment with 3.75, 7.50, and 15.0 *μ*M of **2** for 72 h in HL-60 cells, the total percentage of early apoptotic cells (right low quadrant) and late apoptotic and necrotic cells (right upper quadrant) were increased from 5.41% to 61.34%. The data indicated that compound **2** remarkable induced apoptotic cells death in HL-60 cells in a concentration-dependent manner.

### Effect of mitochondria depolarization by compound 2

**Figure 7:**
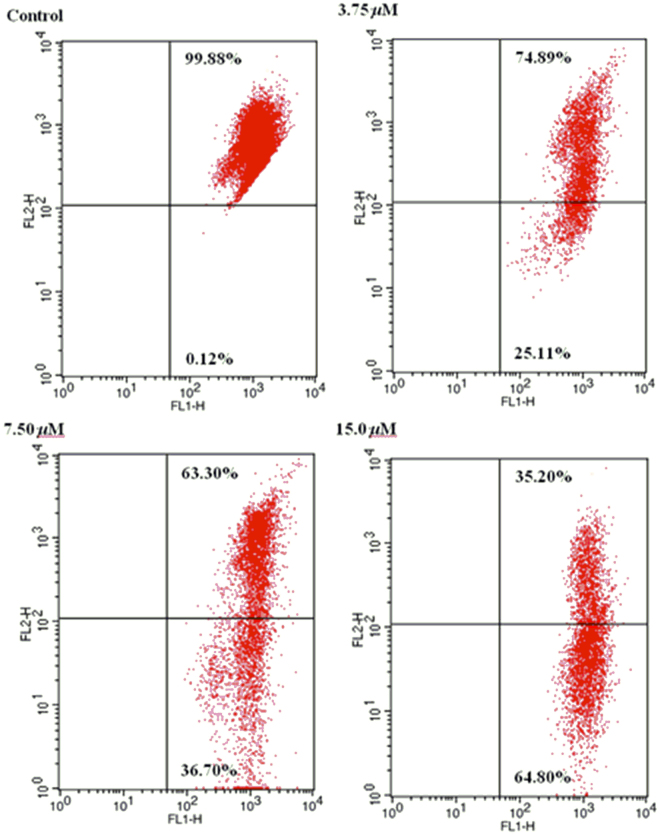
Effect of **2** on the mitochondrial membrane potentials in HL-60 cells.

The destruction of mitochondrial membrane potential is widely considered to be a crucial event in the process of cell apoptosis^15,16^. In order to further research the apoptosis induced effect of compound **2**, the fluorescent probe JC-1 was carried out to detect the changes of mitochondrial membrane potential. JC-1 (a kind of lipophilic cationic dye) can pass the plasma membrane into cells and accumulate in mitochondria. Meanwhile, membrane potential and degree of accumulation of JC-1 in mitochondria exist close contact. Normal cells, normal membrane potential, at the higher concentration of dye JC-1, with the dye aggregation, fluorescence emission gradually changes red. While apoptotic cells, the mitochondrial transmembrane potential depolarization, the monomer of JC-1 is formed, and fluorescence emission changes from red to green. Therefore, it can easily be used to test the changes of mitochondrial membrane potential by detecting the changes of fluorescence color. HL-60 cells dealt with compound **2** at 3.75, 7.50, and 15 *μ*M for 72 h respectively were stained with JC-1, meanwhile untreated cells was used as control. The percentage of green fluorescence increased (0.12%, 25.11%, 36.70%, and 64.80%) in a concentration-dependent manner was observed in Fig. 7. The results demonstrated that **2** could induce apoptosis in HL-60 cells through mitochondrial-related pathway.

## Methods

### General experimental procedures

Optical rotations were measured on Rudolph Autopol-V digital polarimeter and Jasco P-2000 polarimeter. UV spectra were recorded using a Shimadzu UV-2201 spectrometer. IR spectra were recorded on a Bruker IFS-55 spectrometer (using a KBr disk method). ECD spectra were measured on Bio-logic MOS 450 spectropolarimeter. 1D and 2D NMR spectra were acquired with Bruker ARX-300 and AV-600 NMR spectrometers using solvent signals (CDCl_3_: *δ*_H_ 7.26/*δ*_C_ 77.16; CD_3_OD: *δ*_H_ 3.31/*δ*_C_ 49.00), with tetramethylsilane (TMS) as an internal standard. HRESIMS data were obtained using Bruker micro-TOFQ-Q mass spectrometer. LC-MS analysis was performed with Thermo Fisher LCQ Fleet Ion Trap LC/MS^n^, and ESI-IT-MS spectra were performed on a Thermo LCQ Advantage MAX Fleet mass spectrometer. A Shimadzu LC-6 AD equipped with a SPD-20A (UV/DAD) detector was used for HPLC. The chiral HPLC isolation was accomplished on Daicel Chiralpak IB column (4.6 × 250 mm, 5 *μ*m; Daicel Chemical Ltd, Tokyo, Japan). Column chromatography (CC) were performed with silica (100–200 and 200–300 mesh, Qingdao Haiyang Chemical Co., Ltd., Qingdao, China), neutral alumina (100–200 mesh, Sinopharm Chemical Reagent Co. Ltd., Shanghai, China), ODS (50 *µ*m, YMC Co. Ltd., Kyoto, Japan), and Sephadex LH-20 (GE Healthcare, Sweden). TLC analyses were carried out with glass plate precoated silica gel (GF_254_, Qingdao Haiyang Chemical Co., Ltd., Qingdao, China). Spots were visualized under UV light or by spraying with 10% H_2_SO_4_ in 95% EtOH followed by heating or with bismuth potassium iodide solution.

### Plant materials

The plant material was purchased from Anguo Medicines Ltd (Hebei), China, in November 2013, and was identified as the aerial parts of *Macleaya cordata* (Willd.) R. Br. by Prof. Jincai Lu (School of Traditional Chinese Materia Medica, Shenyang Pharmaceutical University, Shenyang, China). The voucher sample (BLH-20131108) was deposited in the Department of Natural Products Chemistry, Shenyang Pharmaceutical University, Shenyang, China.

### Extraction and isolation

The air-dried aerial parts of *M*. *cordata* (40.0 kg) were extracted with 95% ethanol (400 L) under reflux 2 times, and 75% ethanol (1 × 400 L), each time for 2 h, respectively. After the solvent was removed under reduced pressure, the crude extract (14.6 kg) was suspended in H_2_O, successively partitioned with CH_2_Cl_2_ and *n*-BuOH, to afford CH_2_Cl_2_, *n*-BuOH and aqueous extracts. Part of the CH_2_Cl_2_ extract (500 g) was subjected to silica gel column chromatography (CC) and eluted with petroleum ether (60–90 °C) –acetone (100:5, 100:10, 100:20, 100:50, 1:1 and 0:100, v/v) to yield six crude fractions (Fr. A– Fr. F). Fr. A was subsequently loaded onto a silica gel column using petroleum ether–EtOAc (100:0, 100:3, 100:5, 100:8, 100:20, v/v) as the eluent to give five subfractions (Fr. A1 –Fr. A5). Fr. A2 was applied to neutral alumina CC eluting with petroleum ether–EtOAc, and further purified by preparative TLC (PTLC) to afford **4** (4.0 mg). Fr. D was further subjected to ODS CC with MeOH–H_2_O (50:50, 60:40, 65:35, 70:30, 80:20, 90:10, v/v) as the mobile phase to give subfractions (Fr. D1-Fr. D6). The residue of Fr. D1 and Fr. D4 after recrystallization was separated by Sephadex LH-20 CC eluting with CH_2_Cl_2_/MeOH (1:1, v/v) to yield total alkaloids (23.9 g) based on TLC analysis. The total alkaloids fraction was further subjected to ODS CC (MeOH–H_2_O, 30:70 to 90:10, v/v). The fraction of 40% methanol elution was recrystallized to give **5** (4.5 mg). The fraction of 75% methanol elution was separated by performing repeated neutral alumina, silica gel, Sephadex LH-20 CC, and PTLC to yield **1** (5.0 mg), and **3** (7.5 mg). Fr. E was subjected to silica gel CC eluted with CH_2_Cl_2_–MeOH (100:0, 200:1, 100:1, 100:2, v/v) to afford four subfractions Fr. E1–Fr. E4. Fr. E1 was subjected to ODS CC eluting with MeOH–H_2_O gradient system, and further recrystallization to yield **6** (30 mg), and then performed on Sephadex LH-20 CC eluting with CH_2_Cl_2_/MeOH (1:1, v/v) to afford **2** (6.0 mg). The *n*-BuOH extract (185 g) was separated on macroporous resin (D101) using EtOH–H_2_O (30:70, 50:50, 70:30, 95:5, v/v) as the eluent to give four subfractions Fr. G–Fr. J. Fr. H was subjected to ODS CC with MeOH–H_2_O gradient eluting, and the part of 20% MeOH eluting was further purified by reversed-phase preparative HPLC (ODS; 5 *μ*m, 250 × 20 mm; MeOH/H_2_O/DEA, 63:37:0.05, v/v/v; flow rate, 5.0 mL/min) to afford **7** (35 mg, *t*_R_ = 45 min).

Chiral separation of **1**, **2**, and **3** was performed on Daicel chiralpak IB column (250 × 4.6 mm), eluted with *n*-hexane–EtOH–DEA (40: 60: 0.1, v/v/v), flow rate 1.0 mL/min to yield **1a** (1.1 mg, t_R_ 5.930 min), **1b** (1.1 mg, *t*_R_ 8.022 min), **2a** (1.5 mg, *t*_R_ 5.813 min), **2b** (1.5 mg, *t*_R_ 12.305 min), **3a** (1.7 mg, *t*_R_ 12.087 min), and **3b** (1.7 mg, *t*_R_ 8.074 min), respectively.

Macleayin F (**1**). White amorphous powder. UV (CH_2_Cl_2_) *λ*_max_ (log *ε*): 227 (4.6), 287 (4.6) nm. IR (KBr) *ν*_max_: 2790, 1656, 1616, 1486, 1462, 941 cm^−1^. (+)-HRESIMS *m/z* 701.2491 [M + H]^+^ (calcd for C_41_H_37_N_2_O_9_, 701.2494).

(+)-Macleayin F (**1a**). ${[\alpha ]}_{{\rm{D}}}^{{\rm{20}}}$ + 274 (*c* 0.07, MeOH); ECD (MeOH) *λmax* (*∆ɛ*) 224 (+9.4), 243 (−22.6), 268 (−3.9), 300 (−7.9) nm.

(−)-Macleayin F (**1b**). ${[\alpha ]}_{{\rm{D}}}^{{\rm{20}}}$ −248 (*c* 0.07, MeOH); ECD (MeOH) *λmax* (*∆ɛ*) 221 (−13.5), 243 (+24.8), 269 (+3.4), 300 (+7.5) nm.

Macleayin G (**2**). White amorphous powder. UV (CH_2_Cl_2_) *λ*_max_ (log *ε*): 229 (4.6), 286 (4.6) nm. IR (KBr) *ν*_max_: 2794, 1652, 1619, 1491, 1458, 938 cm^−1^. (+)-HRESIMS *m/z* 717.2799 [M + H]^+^ (calcd for C_42_H_41_N_2_O_9_, 717.2807).

(+)-Macleayin G (**2a**). ${[\alpha ]}_{{\rm{D}}}^{{\rm{20}}}$ + 238 (*c* 0.10, MeOH); ECD (MeOH) *λ*_max_ (*∆ɛ*) 222 (+15.9), 241 (−30.3), 264 (−6.8), 283 (−14.3) nm.

(−)-Macleayin G (**2b**). ${[\alpha ]}_{{\rm{D}}}^{{\rm{20}}}$ −210 (*c* 0.10, MeOH); ECD (MeOH) *λ*_max_ (*∆ɛ*) 223 (−19.4), 241 (+36.0), 265 (+7.3), 283 (+15.1) nm.

Macleayin H (**3**). White amorphous powder. UV (MeOH) *λ*_max_ (log *ε*): 232 (4.6), 287 (4.6) nm. IR (KBr) *ν*_max_: 2798, 1658, 1617, 1485, 1463, 939 cm^−1^. (+)-HRESIMS *m/z* 701.2485 [M + H]^+^ (calcd for C_41_H_37_N_2_O_9_, 701.2494).

(+)-Macleayin H (**3a**). ${[\alpha ]}_{{\rm{D}}}^{{\rm{20}}}$ + 71 (*c* 0.11, MeOH); ECD (MeOH) *λ*_max_ (*∆ɛ*) 204 (+19.3), 233 (−24.0), 271 (−0.2), 291 (−5.7) nm.

(−)-macleayin H (**3b**). ${[\alpha ]}_{{\rm{D}}}^{{\rm{20}}}$ −94 (*c* 0.11, MeOH); ECD (MeOH) *λ*_max_ (*∆ɛ*) 205 (−17.2), 233 (+19.3), 276 (+0.4), 291 (+6.0) nm.

Sanguinarine (**4**). Red amorphous powder. ^1^H NMR (400 MHz, CD_3_OD) *δ*_H_: 9.97 (1 H, s, H-6), 8.67 (1 H, d, *J* = 8.8 Hz, H-10), 8.57 (1 H, d, *J* = 8.9 Hz, H-11), 8.25 (1 H, d, *J* = 8.9 Hz, H-12), 8.18 (1 H, s, H-4), 7.98 (1 H, d, *J* = 8.8 Hz, H-9), 7.60 (1 H, s, H-1), 6.54 (2 H, s, -OCH_2_O-2,3), 6.28 (2 H, s, -OCH_2_O-7,8), 4.97 (3 H, s, *N*-CH_3_). ^13^C NMR (150 MHz, CD_3_OD) *δ*_C_: 107.0 (C-1), 150.8 (C-2), 150.9 (C-3), 105.0 (C-4), 122.0 (C-4a), 132.9(C-4b), 52.8 (*N*-CH_3_), 151.0 (C-6), 111.4 (C-6a), 148.3 (C-7), 149.6 (C-8), 121.3 (C-9), 118.3 (C-10), 129.2 (C-10a), 127.7 (C-10b), 119.7 (C-11), 133.3 (C-12), 134.3 (C-12a), 104.3 (-OCH_2_O-2,3), 106.5 (-OCH_2_O-7,8).

Chelerythrine (**5**). Yellow amorphous powder. ^1^H NMR (400 MHz, CD_3_OD) *δ*_H_: 9.99 (1 H, s, H-6), 8.71 (1 H, d, *J* = 9.0 Hz, H-10), 8.68 (1 H, d, *J* = 9.2 Hz, H-11), 8.24 (1 H, d, *J* = 9.0 Hz, H-9), 8.23 (1 H, d, *J* = 9.2 Hz, H-12), 8.21 (1 H, s, H-4), 7.59 (1 H, s, H-1), 6.28 (2 H, s, -OCH_2_O-2,3), 4.30 (3 H, s, 7-OCH_3_), 4.15 (3 H, s, 8-OCH_3_), 5.01 (3 H, s, *N*-CH_3_). ^13^C NMR (100 MHz, CD_3_OD) *δ*_C_: 107.1 (C-1), 151.8 (C-2), 150.8 (C-3), 105.1 (C-4), 121.9 (C-4a), 132.6 (C-4b), 52.9 (*N*-CH_3_), 152.1 (C-6), 119.9 (C-6a), 147.6 (C-7), 151.8 (C-8), 127.5 (C-9), 121.0 (C-10), 130.2 (C-10a), 127.2 (C-10b), 119.5 (C-11), 132.7 (C-12), 134.4 (C-12a), 104.3 (-OCH_2_O-2,3), 62.8 (7-OCH_3_), 57.6 (8-OCH_3_).

Protopine (**6**). Colourless tetragonal crystal (CH_2_Cl_2_: MeOH = 1:1). ^1^H NMR (400 MHz, CDCl_3_) *δ*_H_: 6.90 (1 H, s, H-1), 6.69 (1 H, d, *J* = 7.8 Hz, H-12), 6.66 (1 H, d, *J* = 7.8 Hz, H-11), 6.64 (1 H, s, H-4), 5.95 (2 H, s, -OCH_2_O-2,3), 5.92 (2 H, s, -OCH_2_O-9,10), 3.78 (2 H, br s, H-13), 3.58 (2 H, br s, H-8), 2.2–3.2 (4 H, br s, H-5, 6), 1.91 (3 H, s, *N*-CH_3_). ^13^C NMR (100 MHz, CDCl_3_) *δ*_C_:108.3 (C-1), 146.5 (C-2), 148.1 (C-3), 110.6 (C-4), 132.9 (C-4a), 31.9 (C-5), 57.9 (C-6), 50.9 (C-8), 118.0 (C-8a),146.0 (C-9), 146.1 (C-10), 106.9 (C-11), 125.2 (C-12), 129.1 (C-12a), 46.6 (C-13), 195.1 (C-14), 136.3 (C-14a), 101.3 (-OCH_2_O-2,3), 101.0 (-OCH_2_O-9,10), 41.6 (*N*-CH_3_).

Allocryptopine (**7**). White amorphous powder. ^1^H NMR (400 MHz, CDCl_3_) *δ*_H_: 6.95 (1 H, s, H-1), 6.91 (1 H, d, *J* = 8.2 Hz, H-12), 6.80 (1 H, d, *J* = 8.2 Hz, H-11), 6.63 (1 H, s, H-4), 5.94 (2 H, s, -OCH_2_O-2,3), 3.86 (3 H, s, 10-OCH_3_), 3.78 (3 H, s, 9-OCH_3_), 3.73 (2 H, br s, H-13), 3.0−3.4 (2 H, br s, H-8), 2.2−3.0 (4 H, br s, H-5, 6), 1.86 (3 H, s, *N*-CH_3_). ^13^C NMR (100 MHz, CDCl_3_) *δ*_C_: 110.6 (C-1), 146.2 (C-2), 148.2 (C-3), 109.4 (C-4), 136.2 (C-4a), 32.5 (C-5), 57.7 (C-6), 50.3 (C-8), 128.7 (C-8a), 151.7 (C-9), 147.6 (C-10), 110.8 (C-11), 127.9 (C-12), 129.8 (C-12a), 46.5 (C-13), 193.5 (C-14), 133.0 (C-14a), 101.3 (-OCH_2_O-2,3), 60.9 (9-OCH_3_), 55.8 (10-OCH_3_), 41.4 (*N*-CH_3_).

### Antiproliferative activity

The HL-60 (human leukaemia cell lines), MCF-7 (human breast cancer cell lines), A-549 (human lung adenocarcinoma cell lines) used in this study were purchased from America Type Culture Collection, ATCC (Rockville, MD, USA). All were cultured in RPMI-1640 medium (Gibco, New York, NY, USA) supplemented with 100 U/mL penicillin, 100 *μ*g/mL streptomycin, 1 mM glutamine and 10% heat-inactivated fetal bovine serum (Gibco) at 37 °C in humidified atmosphere with 5% CO_2_. Cytotoxic activity was evaluated by the trypan blue method against HL-60, and MTT assay against MCF-7 and A-549.

### Cell cycle study

HL-60 cells in logarithmic growth were plated in 6-well plates and incubated for 24 h, then incubated with different concentrations (0, 3.75, 7.50, 15.0 *μ*M) of **2** (DMSO only as control) at 37 °C for 72 h. The cells were washed with ice-cold PBS buffer, and then collected, fixed with 70% EtOH at −4 °C for 24 h. The fixed cells were washed with ice-cold PBS and then were treated with 100 *μ*L RNase A at 37 °C for 30 min, and finally stained with 400 *μ*L propidium iodide (PI) in the dark at 4 °C for 30 min. The cycle distribution analysis was performed using a flow cytometer (FACS Calibur, Becton-Dickinson, America).

### Cell apoptosis analysis

HL-60 cells in logarithmic growth were plated in 6-well plates and incubated for 24 h, then incubated with different concentrations (0, 3.75, 7.50, 15.0 *μ*M) of **2** (DMSO only as control) at 37 °C for 72 h. The cells were washed twice with ice-cold PBS buffer, and then collected. 500 *μ*L binding buffer suspension cells were added, and finally double stained Annexin V–APC/7-AAD at room temperature for 15 min in the dark. The apoptotic cells were detected to analyze apoptosis by flow cytometry.

### Mitochondrial membrane potential assay

HL-60 cells in logarithmic growth were plates in 6-well and incubated for 24 h, then incubated with different concentrations (0, 3.75, 7.50, 15.0 *μ*M) of **2** (DMSO only as control) at 37 °C for 48 h. The cells were washed with ice-cold PBS buffer, subsequently collected and adjusted the cells concentration to 1 × 10^6^/ml, and finally stained according to the manufacture’s instruction (Keygen, KGA601, Nanjing, China) with JC-1. The percentage of green fluorescence was detected by the flow cytometry to analyze the cells apoptosis and collapsed mitochondrial membrane potentials.

## Electronic supplementary material

Supplementary Information
