# Supplementary material for: LC-MS guided isolation of three pairs of enantiomeric alkaloids from Macleaya cordata and their enantioseparations, antiproliferative activity, apoptosis-inducing property
Source: Sci Rep. 2017 Nov 13;7:15410. doi: 10.1038/s41598-017-15423-4 (PMC5684219; doi:10.1038/s41598-017-15423-4)
Supplement: Supplementary file 1 — Supplementary Information [file 41598_2017_15423_MOESM1_ESM.pdf]

LC-MS guided isolation of three pairs of enantiomeric alkaloids from *Macleaya cordata* and their enantioseparations, antiproliferative activity, apoptosis-inducing property

Chunmei Sai[1,2], Dahong Li[2], Shengge Li[2], Tong Han[2], Yongzhi Guo[2], Zhanlin Li[2] & Huiming Hua[2]

[1]School of Pharmacy, Jining Medical University, Rizhao 276826, Shandong Province, People's Republic of China.

[2]Key Laboratory of Structure-Based Drug Design and Discovery, Ministry of Education, Shenyang Pharmaceutical University, Shenyang 110016, Liaoning Province, People's Republic of China.

Correspondence and requests for materials should be addressed to H.H. (huimhua@163.com), S.C. (saichunmei1980@163.com), L.Z. (lzl1030@hotmail.com)

Contents

1 LC-MS analyses of the crude ethanol extracts 3

2 The key HMBC and NOE correlations of compounds **2** and **3** 5

3. ^{1}H, and ^{13}C NMR data of macleayins A, B, F (**1**), G (**2**), and H (**3**) 5

4. Chiral separation of **1**, **2**, and **3** 7

5. The 1D and 2D NMR spectra of **1** 9

Figure S7 ^{1}H NMR spectrum for **1** in $CDCl_3$ 9

Figure S8 ^{13}C NMR spectrum for **1** in $CDCl_3$ 9

Figure S9 HSQC spectrum for **1** in $CDCl_3$ 10

Figure S10 HMBC spectrum for **1** in $CDCl_3$ 10

Figure S11 ^{1}H-^{1}H COSY spectrum for **1** in $CDCl_3$ 11

Figure S12 NOESY spectrum for **1** in $CDCl_3$ 11

6 Figure S13 HRESIMS and HRESIMS/MS of **1** 12

7. Figure S14 IR spectrum of **1** 13

8 Figure S15 UV spectrum of **1** in CH_2Cl_2 13

9 The 1D and 2D NMR spectra of **2** 14

Figure S16 ^{1}H NMR spectrum for **2** in $CDCl_3$ 14

Figure S17 ^{13}C NMR spectrum for **2** in $CDCl_3$ 14

Figure S18 HSQC spectrum for **2** in $CDCl_3$ 15

Figure S19 HMBC spectrum for **2** in $CDCl_3$ 15

Figure S20 ^{1}H-^{1}H COSY spectrum for **2** in $CDCl_3$ 16

Figure S21 NOESY spectrum for **2** in $CDCl_3$ 16

10 Figure S22 HRESIMS and HRESIMS/MS of **2** 17

11 Figure S23 IR spectrum of **2** 18

13 The 1D and 2D NMR spectra of **3** 19

Figure S25 ^{1}H NMR spectrum for **3** in $CDCl_3$ 19

Figure S26 ^{1}H NMR spectrum for **3** in DMSO 19

Figure S27 ^{13}C NMR spectrum for **3** in $CDCl_3$ 20

Figure S28 ^{13}C NMR spectrum for **3** in DMSO 20

Figure S29 HSQC spectrum for **3** in DMSO 21

Figure S30 HMBC spectrum for **3** in DMSO 21

Figure S31 ^{1}H-^{1}H COSY spectrum for **3** in DMSO 22

Figure S32 NOESY spectrum for **3** in DMSO 22

14 Figure S33 HRESIMS and HRESIMS/MS of **3** 23

15 Figure S34 IR spectrum of **3** 24

16 Figure S35 UV spectrum of **3** 24

1 LC-MS analyses of the crude ethanol extracts

LC-MS analysis was performed with Thermo Fisher LCQ Fleet Ion Trap LC/MS^n. Chromatographic separation was achieved on a Unitary C_{18} column (150 × 2.1 mm i.d., 5 μm, ACCHROM). The mobile phase with a flow rate of 0.2 mL/min consisted of solvent A (H_2O with 0.1% $NH_3 \cdot H_2O$) and solvent B (acetonitrile). A binary gradient elution was performed as follows:

Time (min)	A%	B%
0.00	40	60
10.00	30	70
30.00	30	70
30.10	10	90
45.00	10	90
45.10	40	60
55.00	40	60

The injection volume was 2 μL for each LC-MS analysis.

MS experiments were achieved in automatic pattern in positive ion modes. Scan range is *m/z* 100–2000 for MS.

Figure S1 LC-MS analysis chromatogram of the crude ethanol extracts

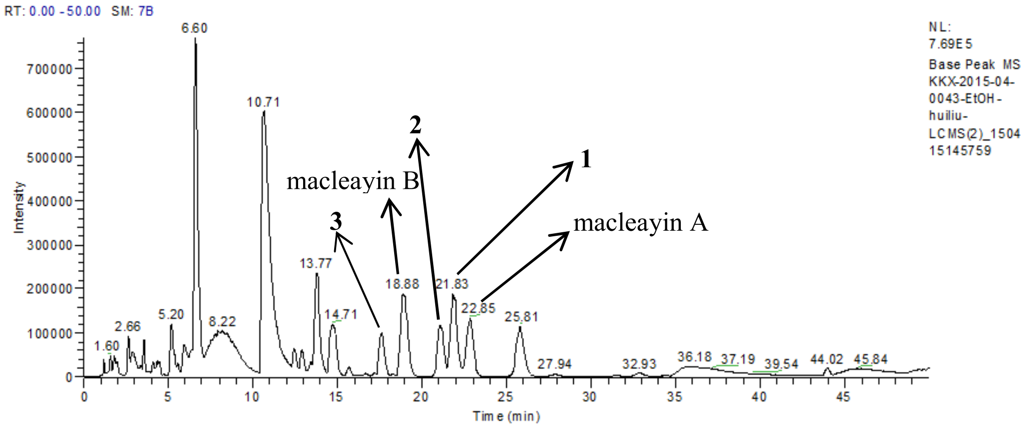

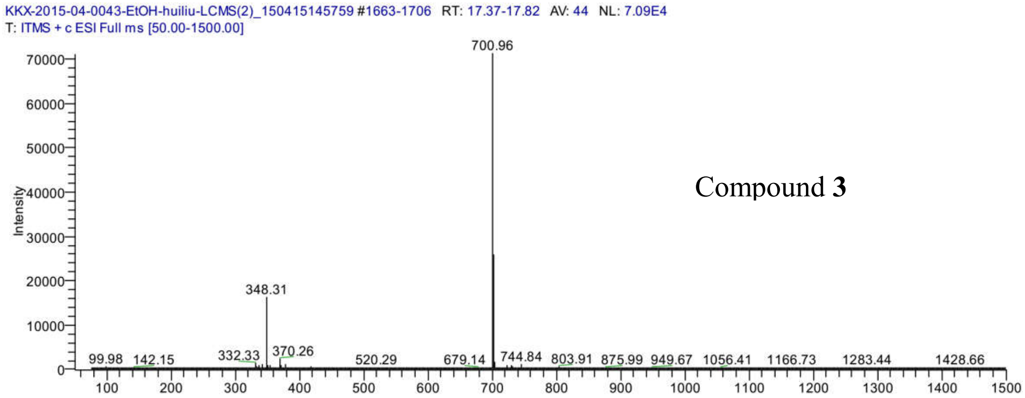

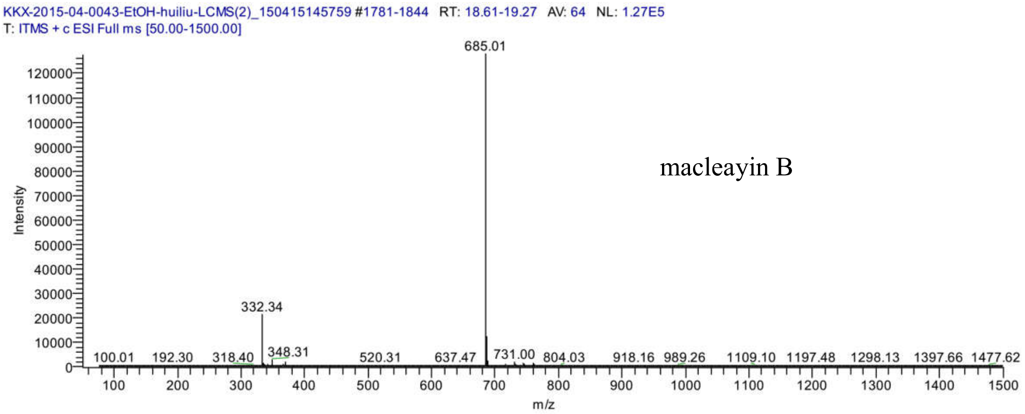
KKX-2015-04-0043-EtOH-huiliu-LCMS(2)_150415145759 #1781-1844 RT: 18.61-19.27 AV: 64 NL: 1.27E5
T: ITMS + c ESI Full ms [50.00-1500.00]
macleayin B
685.01
332.34
100.01 192.30 318.40 348.31 520.31 637.47 731.00 804.03 918.16 989.26 1109.10 1197.48 1298.13 1397.66 1477.62
Intensity
m/z

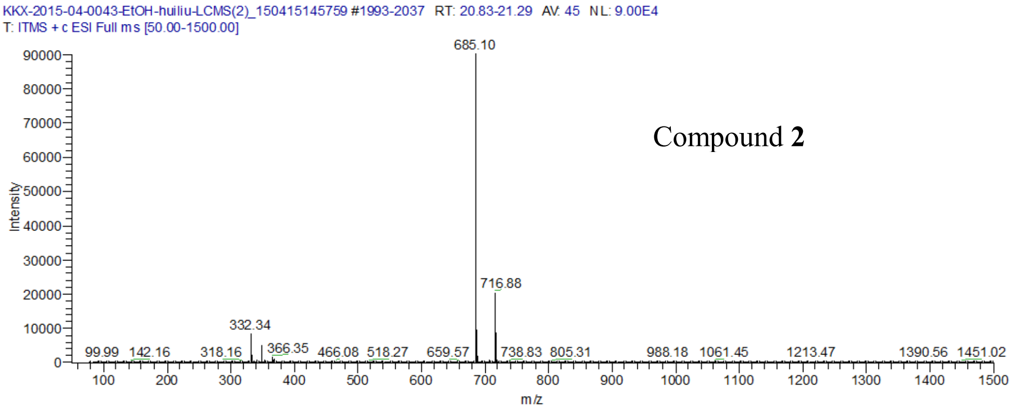
KKX-2015-04-0043-EtOH-huiliu-LCMS(2)_150415145759 #1993-2037 RT: 20.83-21.29 AV: 45 NL: 9.00E4
T: ITMS + c ESI Full ms [50.00-1500.00]
Compound **2**
685.10
716.88
332.34
99.99 142.16 318.16 366.35 466.08 518.27 659.57 738.83 805.31 988.18 1061.45 1213.47 1390.56 1451.02
Intensity
m/z

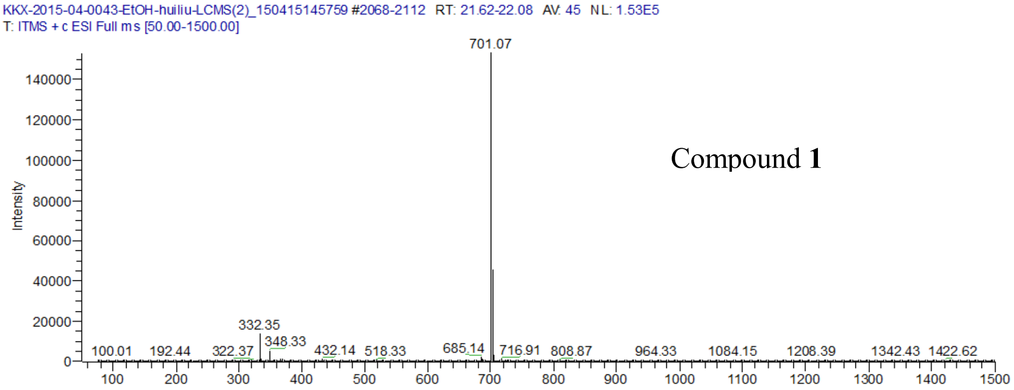
KKX-2015-04-0043-EtOH-huiliu-LCMS(2)_150415145759 #2068-2112 RT: 21.62-22.08 AV: 45 NL: 1.53E5
T: ITMS + c ESI Full ms [50.00-1500.00]
Compound **1**
701.07
332.35
348.33
100.01 192.44 322.37 432.14 518.33 685.14 716.91 808.87 964.33 1084.15 1208.39 1342.43 1422.62
Intensity

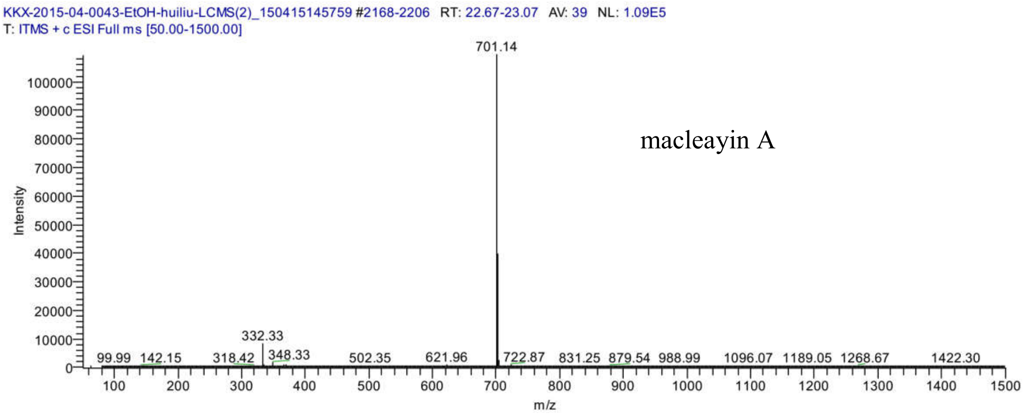
KKX-2015-04-0043-EtOH-huiliu-LCMS(2)_150415145759 #2168-2206 RT: 22.67-23.07 AV: 39 NL: 1.09E5
T: ITMS + c ESI Full ms [50.00-1500.00]
macleayin A
701.14
332.33
99.99 142.15 318.42 348.33 502.35 621.96 722.87 831.25 879.54 988.99 1096.07 1189.05 1268.67 1422.30
Intensity
m/z

2 The key HMBC and NOE correlations of compounds 2 and 3

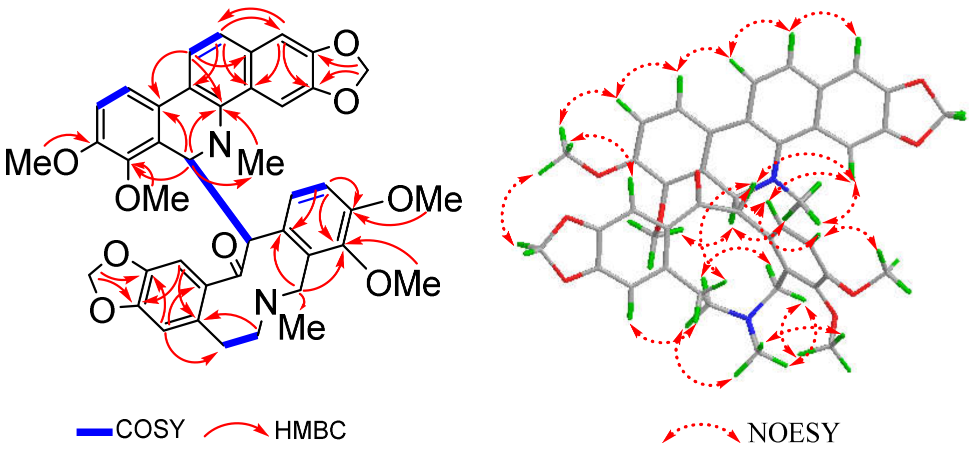

Figure S2 HMBC, ^{1}H-^{1}H COSY, and NOESY correlations of **2**

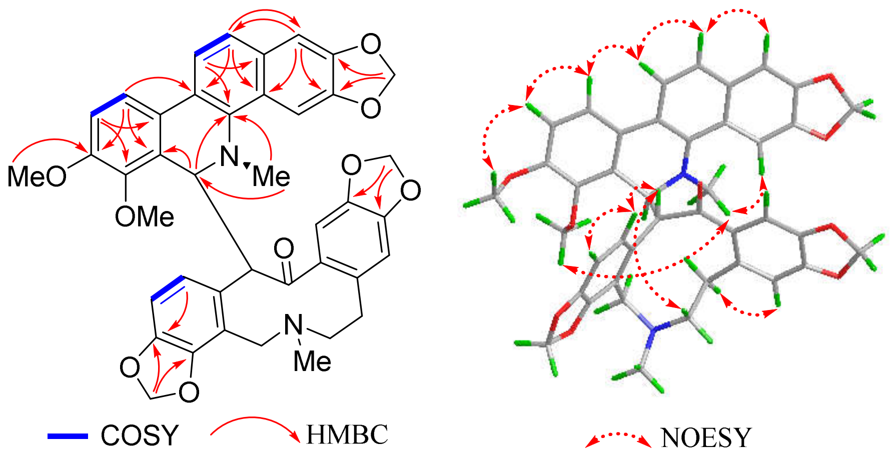

Figure S3 HMBC, ^{1}H-^{1}H COSY, and NOESY correlations of **3**

3. ^{1}H, and ^{13}C NMR data of macleayins A, B, F (1), G (2), and H (3)

Table S1 ^{1}H NMR data of macleayins A, B, F, G, and H

Position	macleayin A[a]	maleayin F[a]	macleayin G[a]	macleayin B[a]	macleayin B[b]	macleayin H[a]	macleayin H[b]
	δ_H (mult., J in Hz)						
1	7.01 (s)	7.05 (s)	7.03 (s)	7.01 (s)	7.21 (s)	7.01 (s)	7.20 (s)
4	6.61 (s)	6.85 (br s)	6.77 (s)	5.35 (br s)	6.68 (br s)	5.29 (m)	6.62 (br s)
6	4.81 (d, 9.3)	5.13 (d, 9.0)	5.14 (d, 10.3)	-[c]	5.24 (br s)	-[c]	5.15 (d, 8.8)
9	6.57 (br s)	6.68(br d, 7.3)	6.66 (br s)	6.72 (d, 8.0)	6.83 (d, 8.1)	6.84 (d, 8.4)	6.97 (d, 8.5)
10	7.12 (brd, 7.9)	7.23(br d, 7.3)	7.23 (br s)	7.21 (d, 8.0)	7.34 (d, 8.1)	7.39 (d, 8.4)	7.49 (d, 8.5)
11	7.68 (d, 8.5)	7.61 (d, 8.5)	7.63 (d, 8.5)	7.74 (d, 8.3)	7.57 (d, 8.5)	7.78 (d, 8.4)	7.85 (d, 8.0)
12	7.46 (d, 8.5)	7.46 (d, 8.5)	7.45 (d, 8.5)	7.49 (d, 8.3)	7.53 (br s)	7.48 (d, 8.4)	7.56 (d, 8.0)
1′	-[c]	6.83 (s)	6.92 (s)	-[c]	-[c]	-[c]	-[c]
4′	6.23 (s)	6.21 (s)	6.19 (s)	5.92 (br s)	6.08 (s)	5.89 (s)	6.07 (s)
5′	2.56 (br s)<br>1.96 (d, 15.0)	2.37–2.31 (m)<br>1.93 (brs)	2.50–2.33 (m)<br>1.86–1.65 (m)	2.30–1.65 (m)	2.28–1.50 (m)	2.23–1.59 (m)	2.27–1.50 (m)

Position	macleayin A[a]	maleayin F[a]	macleayin G[a]	macleayin B[a]	macleayin B[b]	macleayin H[a]	macleayin H[b]
	δ_H (mult., J in Hz)						
6′	2.39 (d, 10.3)<br>1.81 (br s)	2.37–2.31 (m)<br>1.85 (br s)	2.50–2.33 (m)<br>1.86–1.65 (m)	2.30–1.65 (m)	2.28–1.50 (m)	2.23–1.59 (m)	2.27–1.50 (m)
8′	3.07 (d, 13.2)<br>2.32 (br s)	2.78 (brd, 9.1)<br>2.37–2.31 (m)	3.09 (d, 13.3)<br>2.50–2.33 (m)	3.22 (br s)<br>2.98 (br s)	3.03 (d, 13.3)<br>2.82 (br s)	3.21 (br s)<br>3.02 (br s)	3.02 (d, 13.4)<br>2.87 (br s)
11′	7.07 (br d, 8.1)	6.97 (brd, 8.0)	7.07 (d, 8.3)	6.78 (d, 6.6)	6.87 (d, 8.2)	6.81 (br s)	6.84 (d, 8.3)
12′	7.50 (br s)	7.50 (br s)	7.61 (d, 8.3)	7.79 (br s)	7.83 (d, 8.2)	7.83 (br s)	7.62 (br s)
13′	4.51 (br s)	4.59 (d, 9.0)	4.47 (d, 10.3)	5.05 (br s)	4.91 (d, 10.1)	5.01 (br s)	4.99 (br s)
5-*N*-CH_3	2.49 (s)	2.51 (s)	2.50 (s)	2.57 (s)	2.48 (s)	2.56 (s)	2.45 (s)
7′-*N*-CH_3	1.52 (s)	1.63 (s)	1.49 (s)	1.87 (s)	1.80 (s)	1.89 (s)	1.86 (s)
2,3-OCH_2O-	5.91 (d, 1.2)<br>5.89 (d, 1.2)	5.99 (d, 1.1)<br>5.96 (d, 1.1)	5.93–5.81 (m)	5.99–5.83 (m)	6.14–5.55 (m)	5.99–5.76 (m)	6.07–5.82
7-OMe<br>8-OMe		3.94 (s)<br>3.83 (s)	3.93 (s)<br>3.81 (s)			3.77 (s)<br>3.65 (s)	3.54 (s)<br>3.72 (s)
7,8-OCH_2O-	6.11 (d, 1.5)<br>5.95 (br s)			5.99–5.83 (m)	6.14–5.55 (m)		
2′,3′-OCH_2O-	5.92 (d, 1.4)<br>5.90 (d, 1.4)	5.91 (d, 1.1)<br>5.83 (d, 1.1)	5.93-5.81 (m)	5.99–5.83 (m)	6.14–5.55 (m)	5.99–5.76 (m)	6.07–5.82
9′-OMe<br>10′-OMe	3.46 (s)<br>3.95 (s)		3.50 (s)<br>3.95 (s)				
9′,10′-OCH_2O-		5.87 (d, 1.4)<br>5.82 (d, 1.4)		5.99–5.83 (m)	6.14–5.55 (m)	5.99–5.76 (m)	6.07–5.82

[a] Measured in $CDCl_3$. [b] Measured in DMSO-d_6. ^{1}H NMR spectra (δ) were measured at 400 MHz for macleayins B[b], H[a] and at 600 MHz for macleayins A[a], F[a], G[a], B[a], H[b]. The assignments were based on ^{1}H–^{1}H COSY, NOESY, HSQC, and HMBC experiments.
-[c] No signal observed in the ^{1}H NMR spectra.

Table S2 ^{13}C NMR data of macleayins A, B, F, G, and H

Position	macleayin A[a]	macleayin F[a]	macleayin G[a]	macleayin B[a]	macleayin H[a]	macleayin H[b]
	δ_C					
1	104.0	104.2	104.1	104.0	104.0	103.7
2	147.5	147.5	147.4	146.6	147.2	146.7
3	147.8	147.9	148.0	147.5	147.4	147.0
4	100.8	100.8	100.8	101.0	100.5	99.8
4a	127.8	127.6	127.8	127.3	127.1	126.2
4b	139.2	139.4	139.4	139.5	139.5	138.7
6	57.3	58.0	57.8	-[c]	63.8	63.0
6a	115.3	126.7	127.0	114.6	125.6	124.5
7	144.8	146.3	146.3	145.1	146.8	145.9
8	147.0	152.1	152.0	146.9	152.1	151.4
9	107.2	111.7	111.5	107.8	112.6	112.7
10	116.6	116.6	118.5	116.6	118.9	118.7
10a	125.6	125.0	125.0	125.9	125.4	
10b	124.5	124.3	124.5	123.8	124.0	123.4
11	119.8	119.8	119.9	120.0	119.8	119.5
12	124.0	123.8	123.7	124.3	124.2	123.8
12a	130.8	131.0	130.8	130.8	130.9	130.3
1′	111.6	111.7	112.1	110.0	-[c]	-[c]
2′	145.6	145.6	145.4	145.9	-[c]	-[c]
3′	148.6	148.3	147.8	146.1	-[c]	-[c]
4′	110.2	109.8	109.7	110.2	110.8	109.8

Position	macleayin A[a]	macleayin F[a]	macleayin G[a]	macleayin B[a]	macleayin H[a]	macleayin H[b]
	δ_C					
4′a	130.4	133.7	134.1	-[c]	-[c]	-[c]
5′	33.9	33.4	33.7	-[c]	-[c]	-[c]
6′	57.3	56.9	57.2	-[c]	-[c]	-[c]
8′	48.4	48.5	48.3	-[c]	-[c]	-[c]
8′a	130.8	118.5	130.6	-[c]	-[c]	-[c]
9′	147.0	145.0	147.0	147.2	-[c]	145.1
10′	150.6	146.0	150.5	150.1	150.1	149.5
11′	110.8	107.1	110.8	106.7	107.7	106.9
12′	125.4	122.5	125.2	124.05	-[c]	-[c]
12′a	131.5	132.9	132.1	-[c]	-[c]	-[c]
13′	52.7	52.8	52.9	64.4	-[c]	-[c]
14′	-[c]	-[c]	-[c]	-[c]	-[c]	-[c]
14′a	135.1	135.5	135.6	-[c]	-[c]	-[c]
5-*N*-CH_3	40.9	41.1	40.8	42.0	43.1	41.2
7′-*N*-CH_3	41.6	41.7	41.5	43.2	41.4	-[c]
2,3-OCH_2O-	101.4	101.0	101.0	101.0	101.1	101.2
2′,3′-OCH_2O-	100.9	101.1	100.9	101.1	101.6	101.7
7-OMe<br>8-OMe		61.0<br>55.9	60.8<br>55.7		61.1<br>56.1	60.4<br>55.7
7,8-OCH_2O-	101.7			100.6		
9′-OMe<br>10′-OMe	60.9<br>56.1		60.9<br>56.0			
9′,10′-OCH_2O-		100.6		101.6	100.9	100.3

[a] Measured in $CDCl_3$. [b] Measured in DMSO-d_6. ^{13}C NMR spectra (δ) were measured at 100 MHz for macleayins A[a], G[a], B[a], H[a] and at 150 MHz for macleayins F[b] an H[b]. The assignments were based on ^1H–^1H COSY, NOESY, HSQC, and HMBC experiments.
-[c] No signal observed in ^{13}C NMR spectra.

4. **Chiral separation of 1, 2, and 3**

Compounds **1**, **2**, and **3** were separated by Shimadzu LC-20AB with DAD detector SPD-20MA (Shimadzu Corporation Analytical & Measuring Instruments Division, Japan) on a chiral column Daicel Chiralpak IB column (Daicel Chemical Industries, Ltd., Japan). The mobile phase consisted of hexane/EtOH/Diethylamine (40:60:0.1), respectively with a flow rate of 1.0ml/min. The detection wavelength was at 230 nm. Their peak areas are almost identical with a ratio of 1:1.

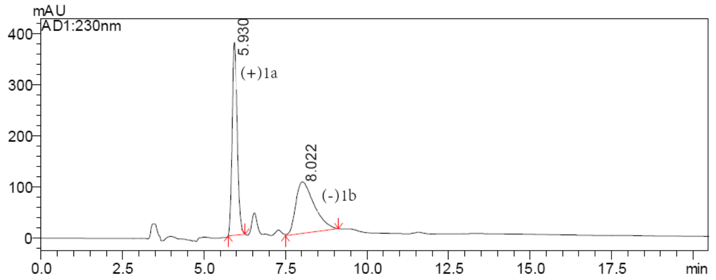

<Column Performance Report>

Peak No.	Time	Area	Area %	Plate number	Tailing	Resolution
1	5.930	4093518	50.6495	6360.824	1.243	--
2	8.022	3988525	49.3505	1960.574	1.629	3.139

Figure S4 The chiral HPLC chromatogram of (±)-macleayin F (**1**)

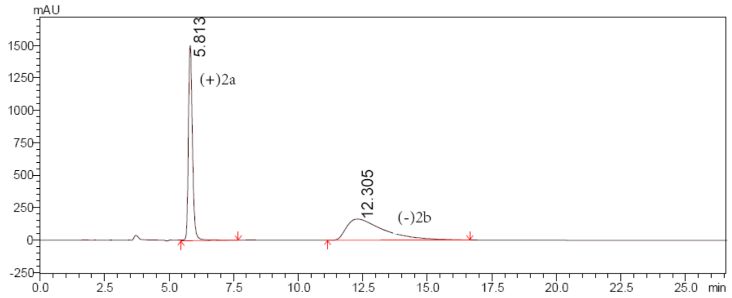

<Column Performance Report>

Peak No.	Time	Area	Area %	Plate number	Tailing	Resolution
1	5.813	16640666	50.3761	6522.886	1.297	--
2	12.305	16392168	49.6239	367.379	2.352	4.546

Figure S5 The chiral HPLC chromatogram of (±)-macleayin G (**2**)

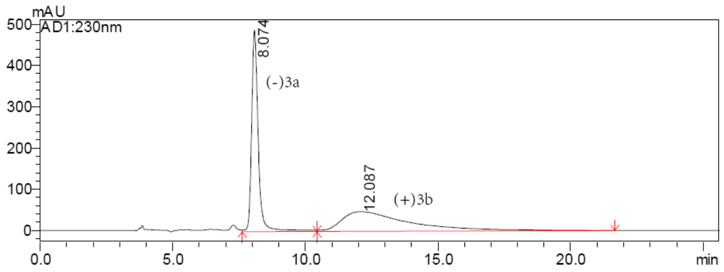

<Column Performance Report>

Peak No.	Time	Area	Area %	Plate number	Tailing	Resolution
1	8.074	8518185	49.8386	5812.851	1.323	--
2	12.087	8573361	50.1614	129.925	--	1.720

Figure S6 The chiral HPLC chromatogram of (±)-macleayin H (**3**)

5. **The 1D and 2D NMR spectra of 1**

Figure S7 ^{1}H NMR spectrum for 1 in $CDCl_3$

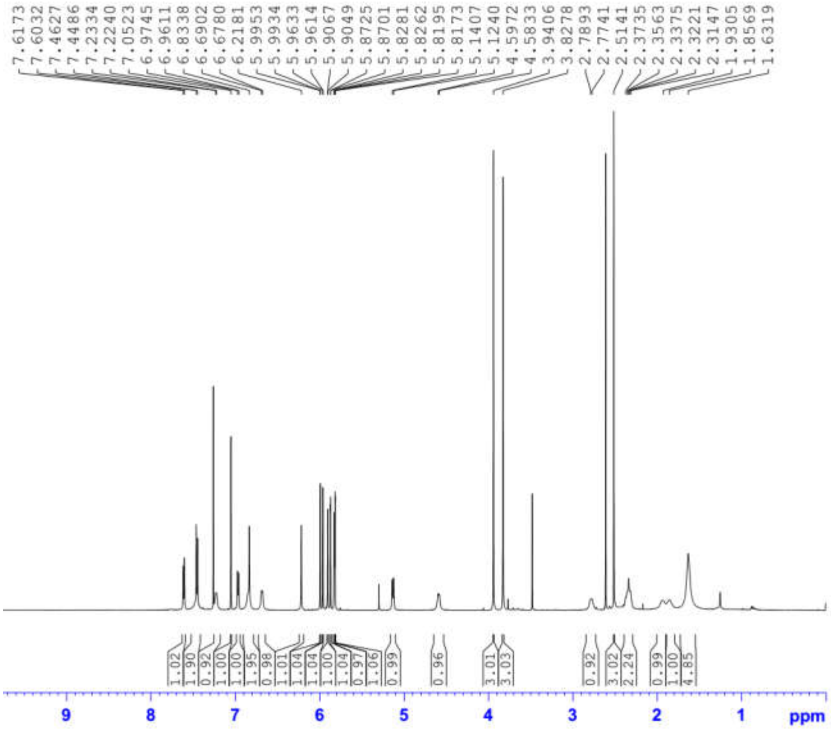

Figure S8 ^{13}C NMR spectrum for 1 in $CDCl_3$

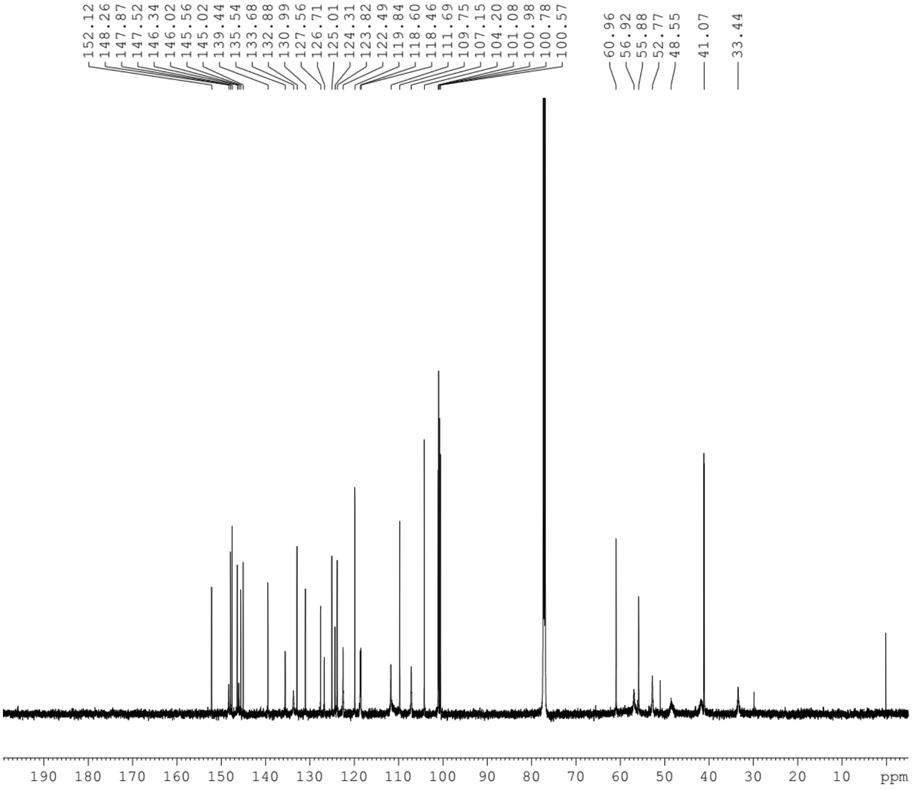

Figure S9 HSQC spectrum for **1** in $CDCl_3$

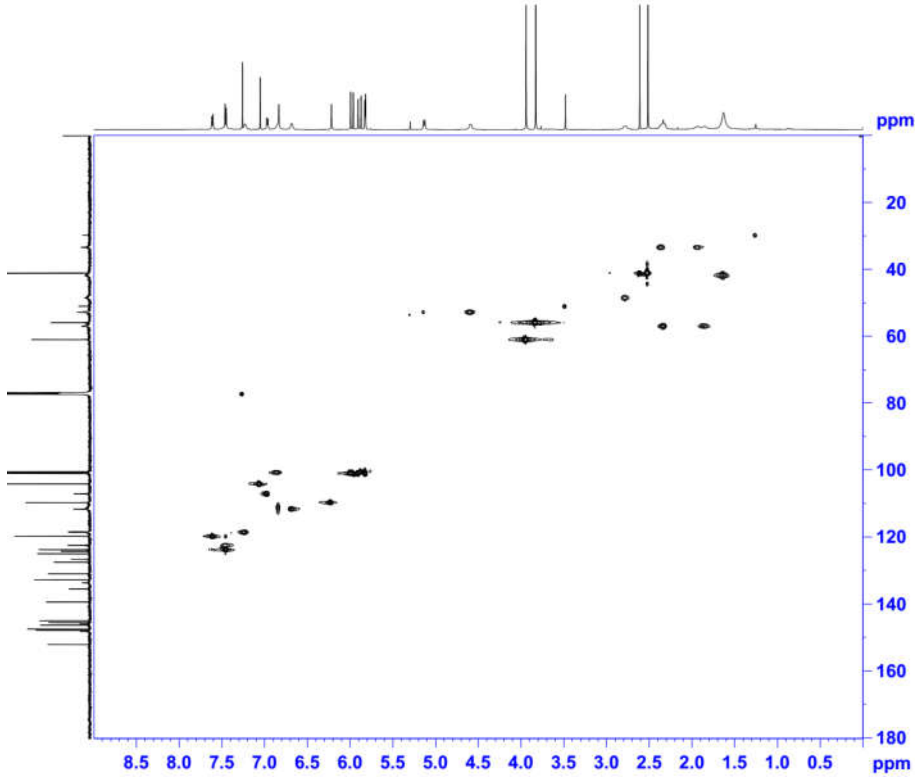

Figure S10 HMBC spectrum for **1** in $CDCl_3$

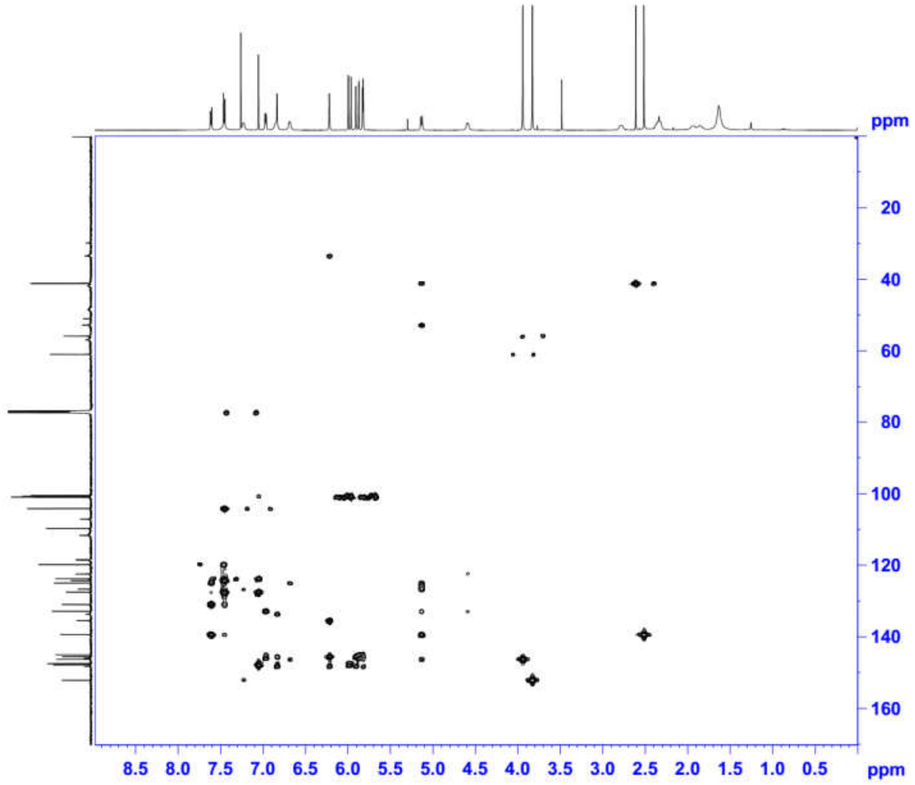

Figure S11 ^{1}H-^{1}H COSY spectrum for **1** in $CDCl_3$

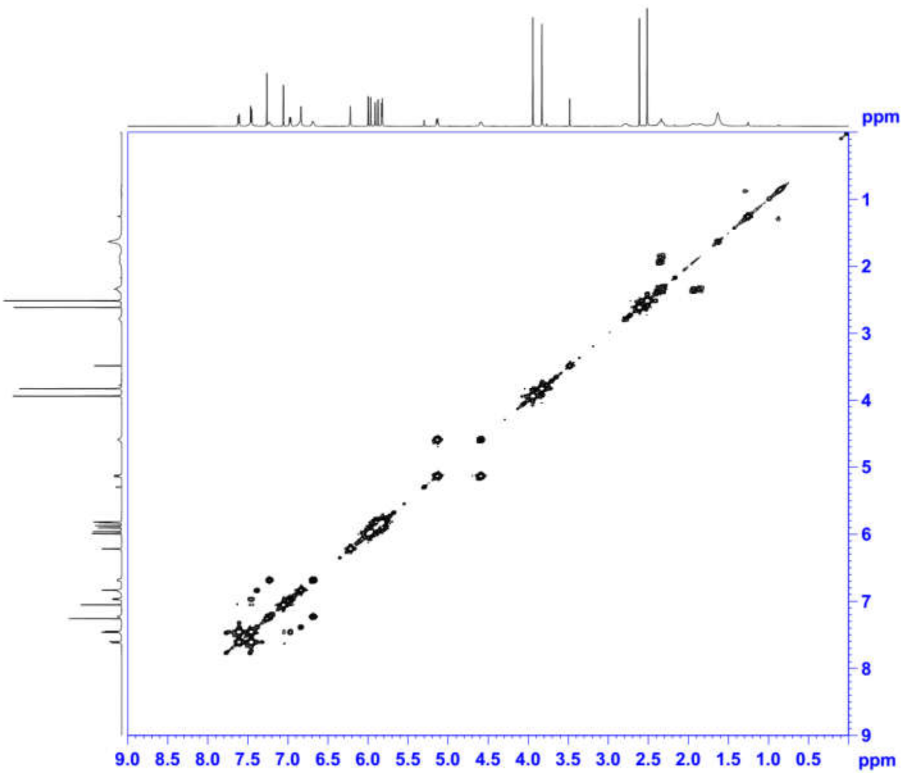

Figure S12 NOESY spectrum for **1** in $CDCl_3$

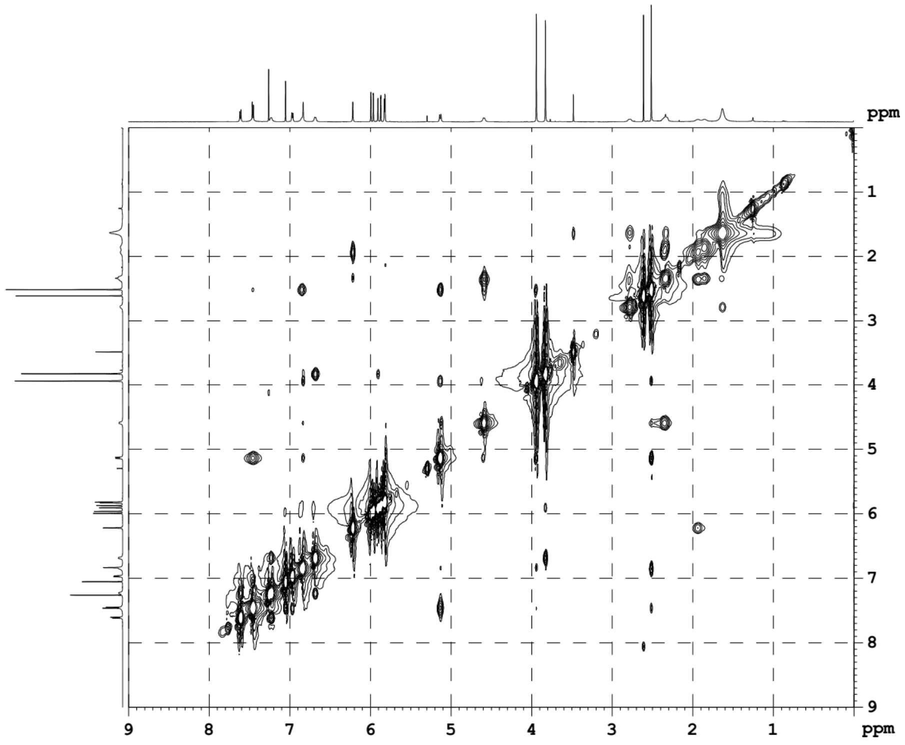

6 **Figure S13 HRESIMS and HRESIMS/MS of 1**

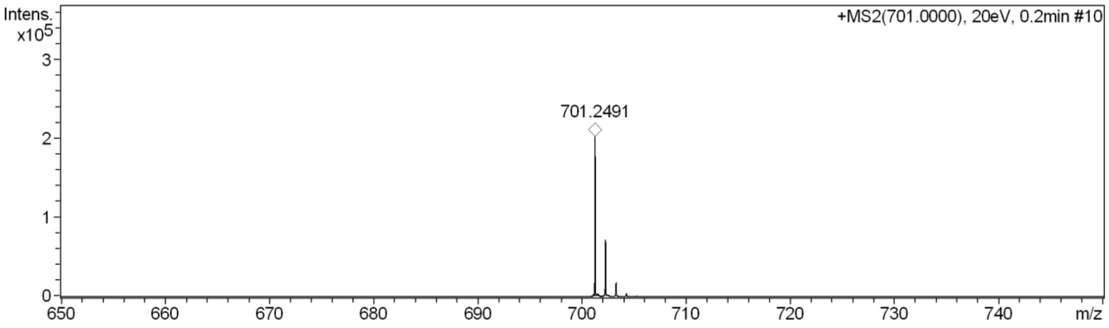

Sum Formula	Sigma	m/z	Err [ppm]	Mean Err [ppm]	Err [mDa]	rdb	N Rule	e⁻
C 41 H 37 N 2 O 9	0.055	701.2494	0.39	3.93	0.27	24.50	ok	even

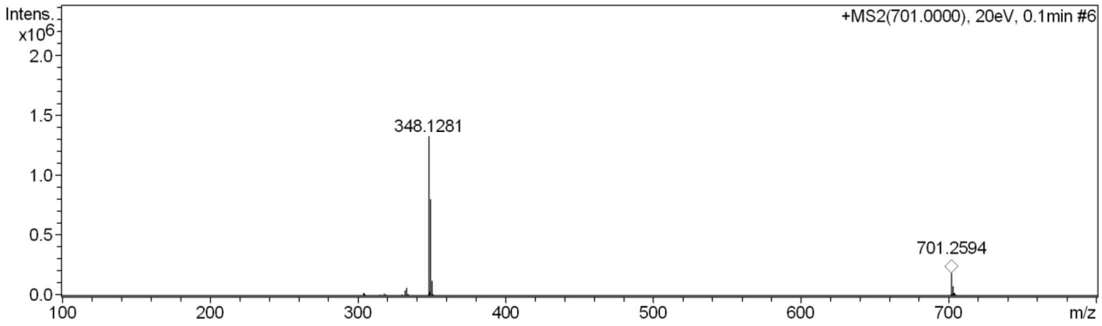

#	m/z	I
1	304.0973	15808
2	305.0991	3220
3	315.0893	3264
4	318.0770	13024
5	332.0923	40844
6	333.0990	59632
7	334.1024	8504
8	347.9692	5708
9	348.1281	1322132
10	348.3627	30900
11	348.5718	6124
12	348.7767	3296
13	349.1345	796788
14	349.2767	4172
15	350.1298	120408
16	351.1321	8868
17	701.2594	184144
18	702.2564	75560
19	703.2554	18496
20	704.2606	3260

7. Figure S14 IR spectrum of 1

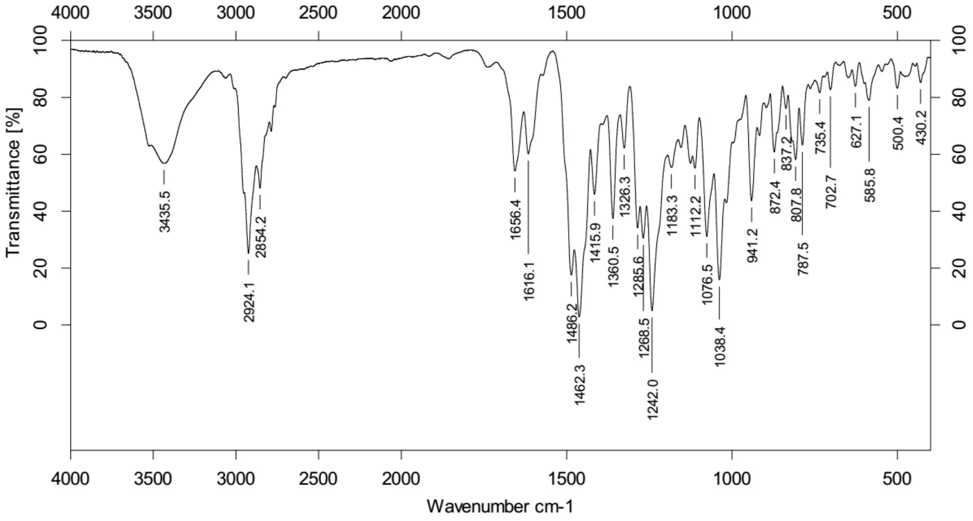

8 Figure S15 UV spectrum of 1 in CH_2Cl_2

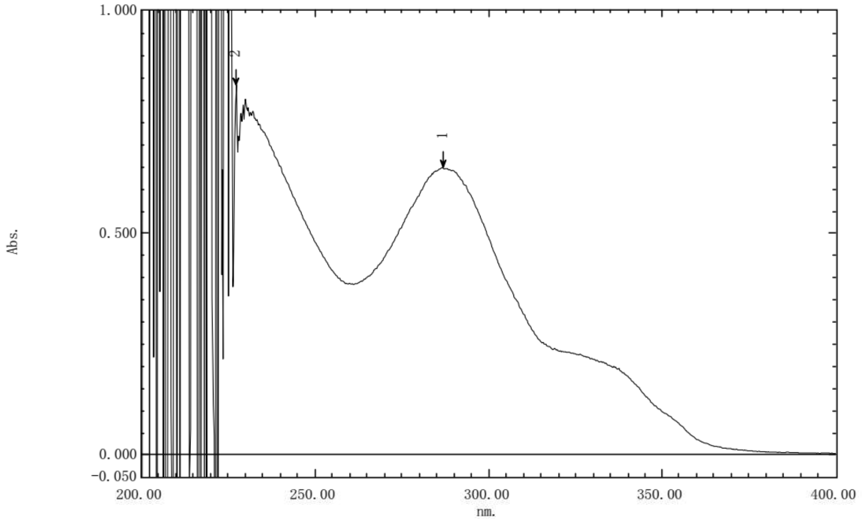

测定属性
波长范围 (nm.): 200.00到400.00
扫描速度: 高速
采样间隔: 0.2
自动采样间隔: 启用
扫描模式: 单一的

试样准备属性
重量: 0.3
体积 : 10
稀释:
光程长: 407
附加信息:

仪器属性
仪器类型: UV-1700
测定方式: 吸收值
狭缝宽: 1.0 nm
光源改变波长: 340.8 nm
S/R 转换: 标准

附件属性
附件: 无

No.	P/V	Wavelength	Abs.	描述
1	↑	286.80	.645	
2	↑	227.40	.832	
3	↑	214.60	4.000	
4	↑	212.00	4.000	
5	↑	200.60	4.000	
6	↓	261.20	.382	
7	↓	226.40	.378	
8	↓	213.80	-.083	
9	↓	206.40	-.602	

9 The 1D and 2D NMR spectra of 2

Figure S16 ^{1}H NMR spectrum for **2** in $CDCl_3$

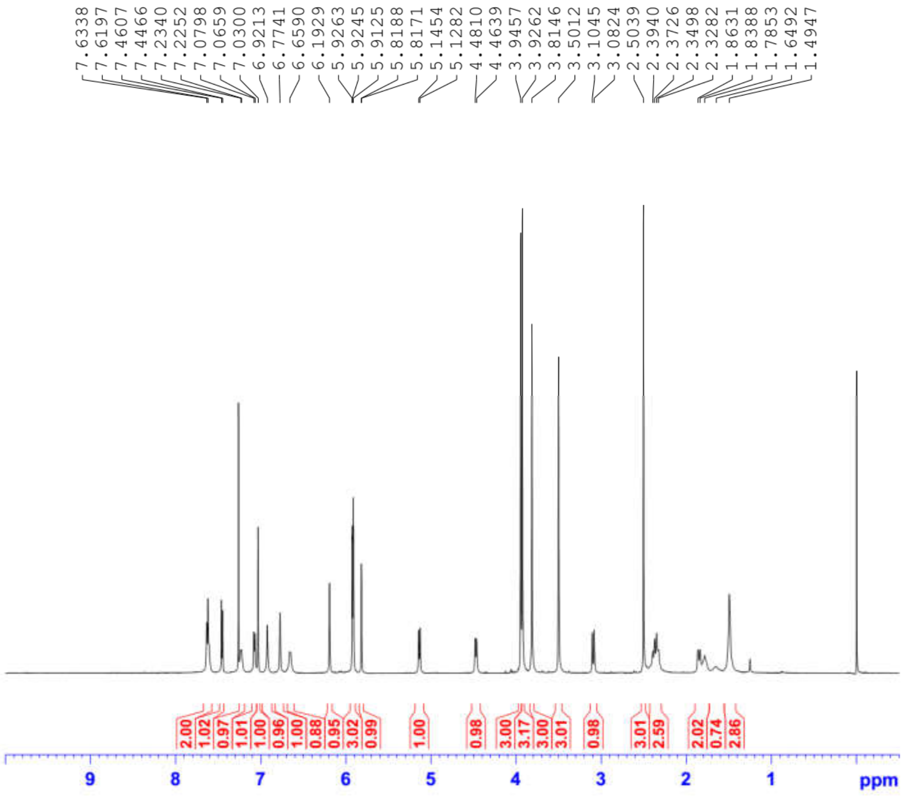

Figure S17 ^{13}C NMR spectrum for **2** in $CDCl_3$

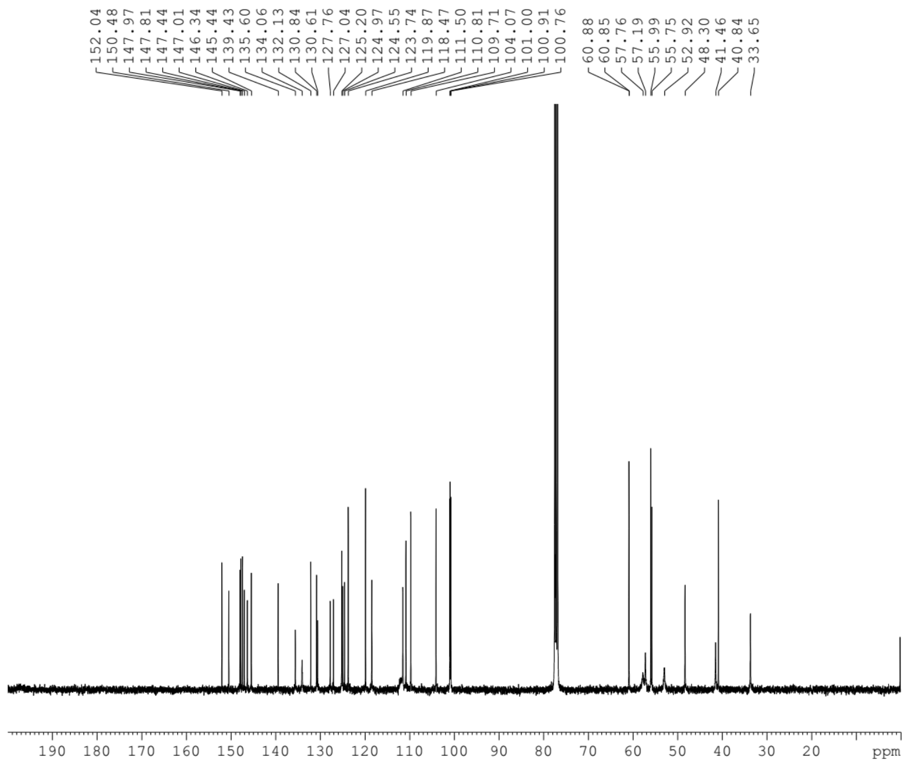

Figure S18 HSQC spectrum for **2** in $CDCl_3$

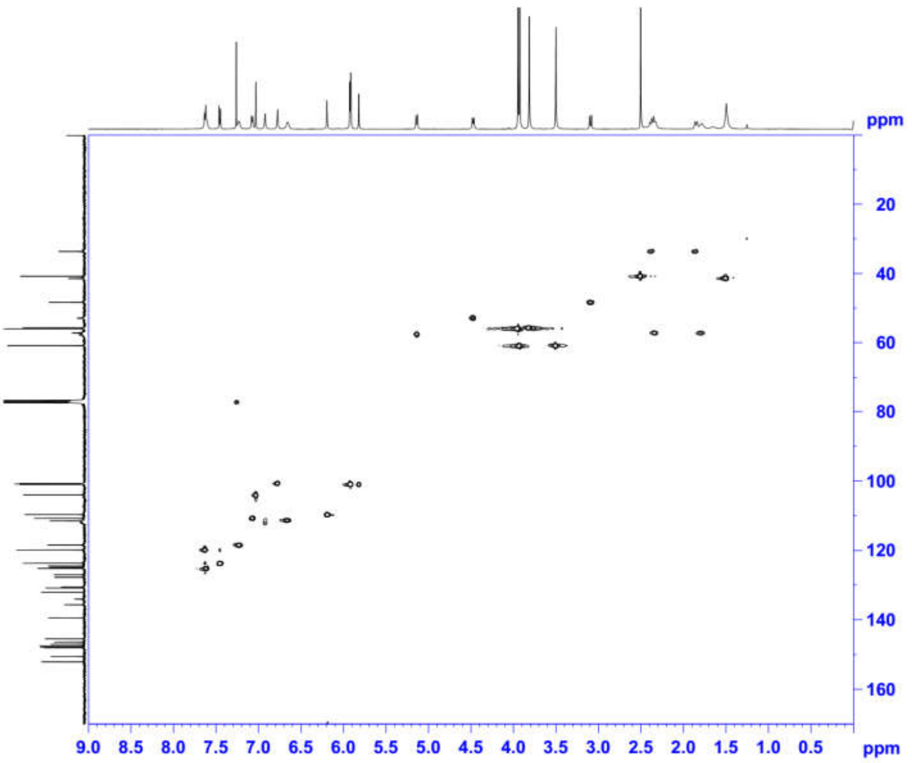

Figure S19 HMBC spectrum for **2** in $CDCl_3$

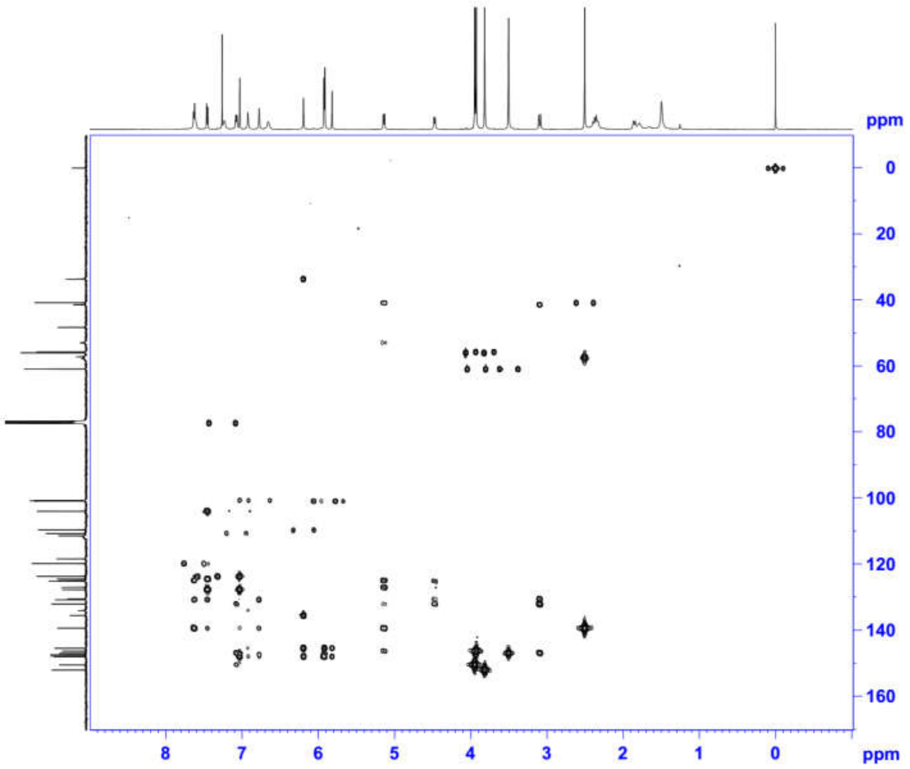

Figure S20 ^{1}H-^{1}H COSY spectrum for **2** in $CDCl_3$

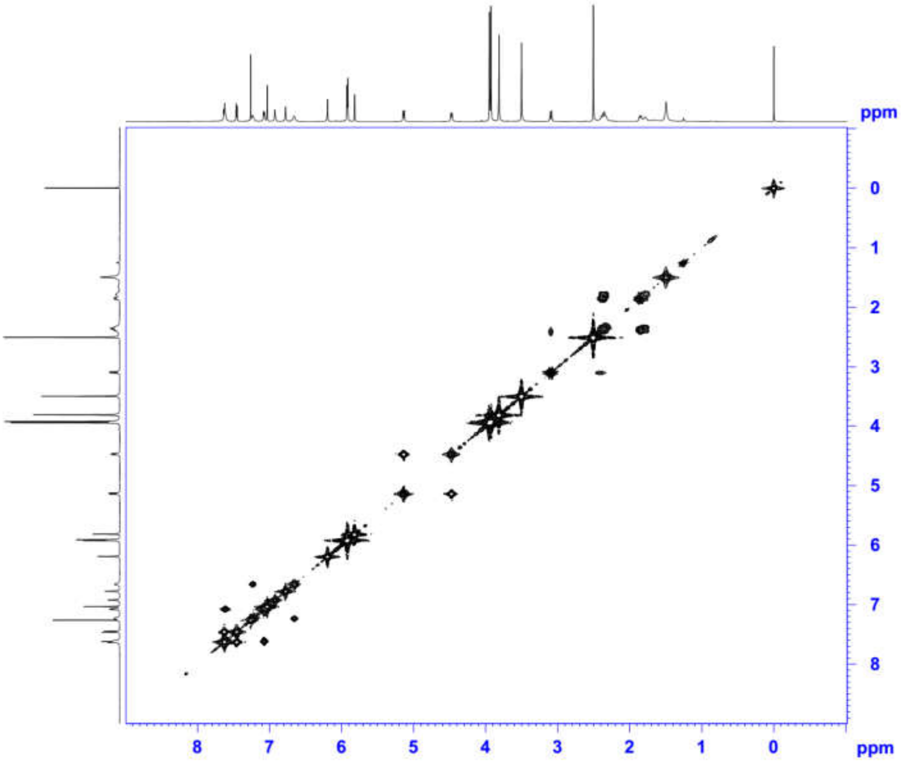

Figure S21 NOESY spectrum for **2** in $CDCl_3$

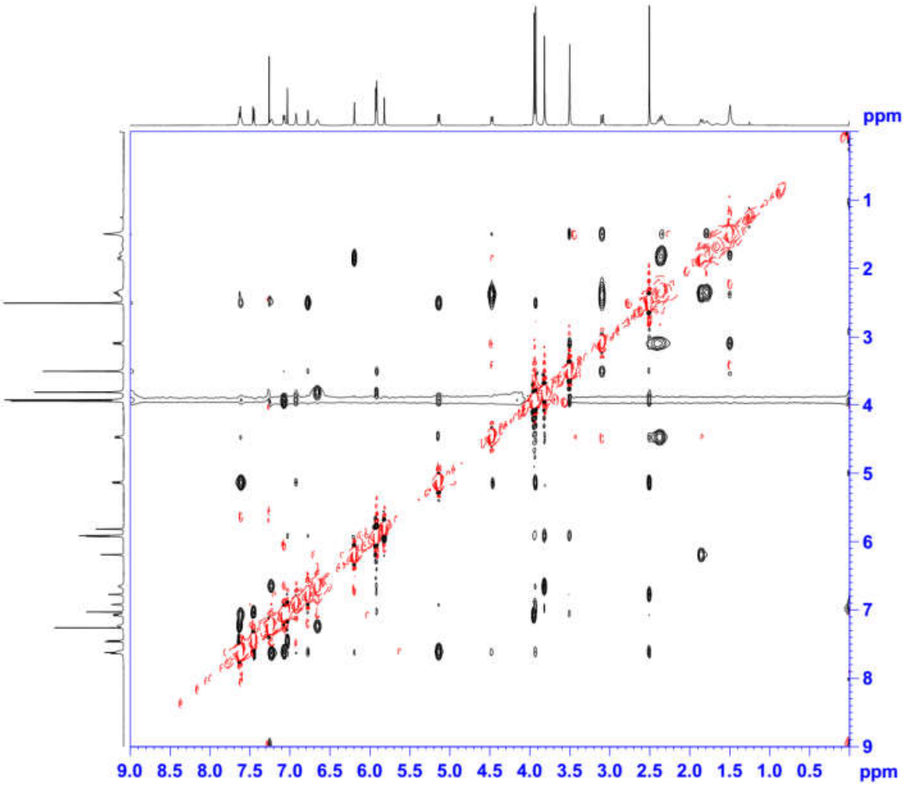

10 Figure S22 HRESIMS and HRESIMS/MS of 2

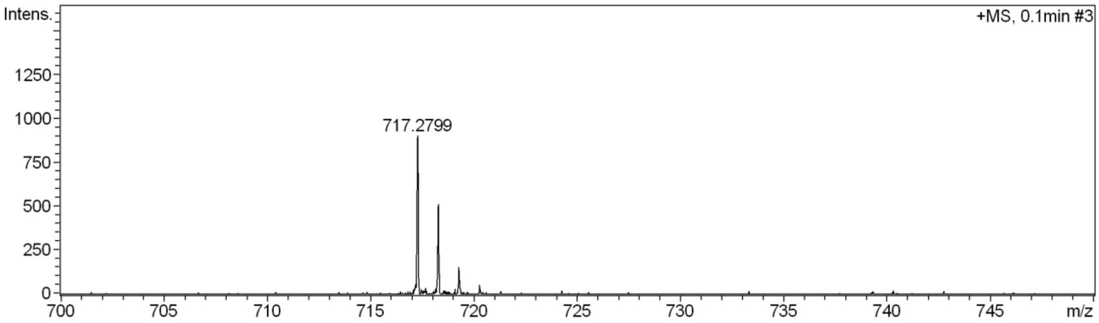

Sum Formula	Sigma	m/z	Err [ppm]	Mean Err [ppm]	Err [mDa]	rdb	N Rule	e⁻
C 42 H 41 N 2 O 9	0.054	717.2807	1.06	0.84	0.76	23.50	ok	even

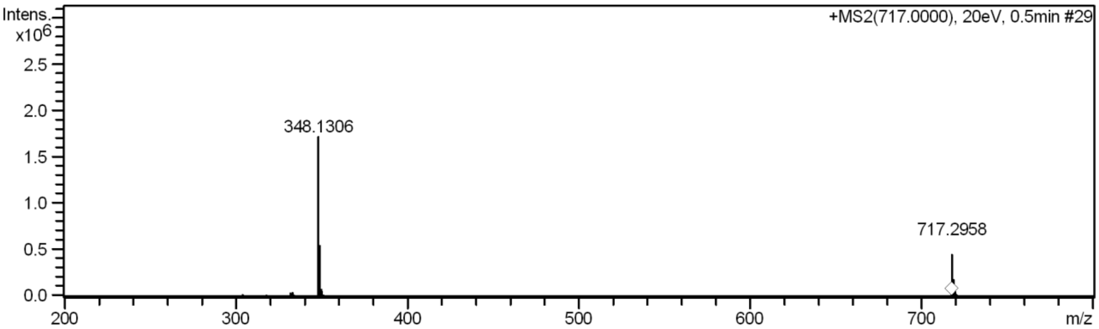

#	m/z	I
1	304.1024	9196
2	315.0952	2252
3	316.0958	2220
4	318.0817	7664
5	332.0956	25004
6	333.1045	32164
7	334.1065	5632
8	347.9841	4392
9	348.1306	1715808
10	348.3099	10384
11	348.3475	11752
12	348.5777	2340
13	349.0230	2052
14	349.1402	542012
15	349.2868	3692
16	350.1346	65484
17	351.1351	5268
18	717.1147	2760
19	717.2958	442256
20	717.5816	4636
21	718.2981	173164
22	718.5462	2376
23	719.2938	42716
24	720.2946	6392

11 Figure S23 IR spectrum of 2

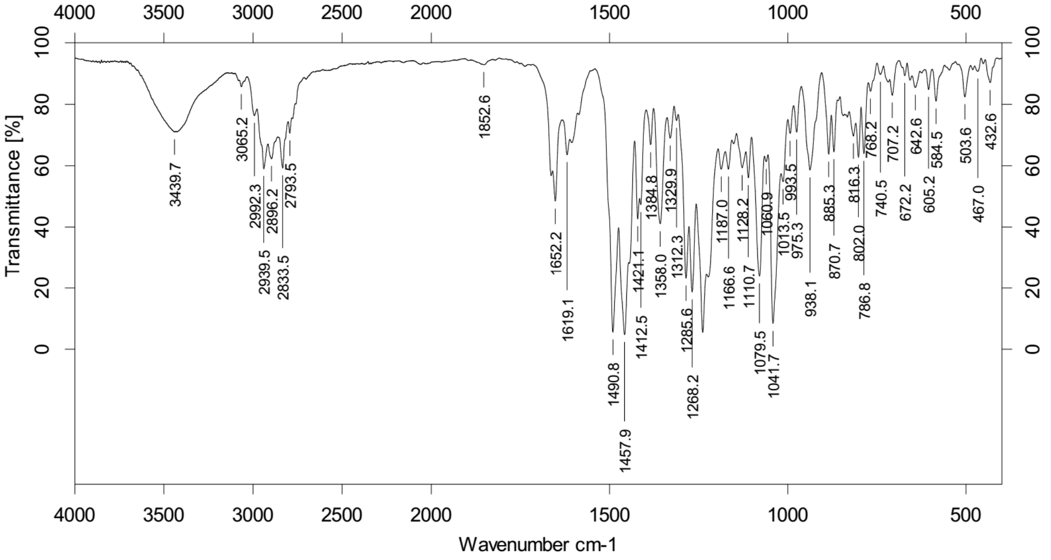

12 Figure S24 UV spectrum of 2 in CH_2Cl_2

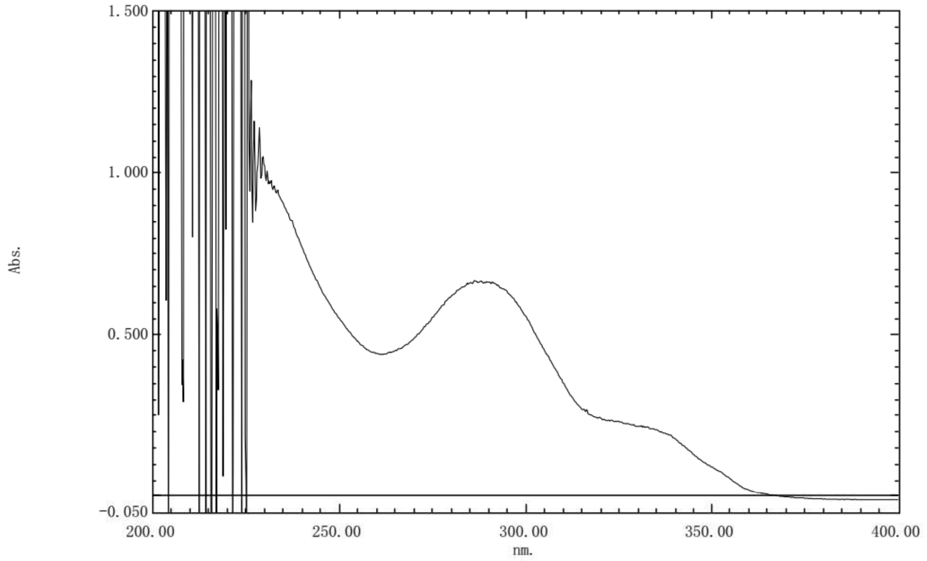

测定属性
波长范围 (nm.): 200.00到400.00
扫描速度: 高速
采样间隔: 0.2
自动采样间隔: 启用
扫描模式: 单一的

试样准备属性
重量: 0.3
体积 : 10
稀释:
光程长: 407
附加信息:

仪器属性
仪器类型: UV-1700
测定方式: 吸收值
狭缝宽: 1.0 nm
光源改变波长: 340.8 nm
S/R 转换: 标准

No.	P/V	Wavelength	Abs.	描述
1	↑	286.40	.667	
2	↑	228.60	1.138	
3	↑	222.00	4.000	
4	↑	213.20	4.000	
5	↑	208.60	4.000	
6	↑	204.60	4.000	
7	↓	261.20	.438	
8	↓	225.20	-.321	
9	↓	221.40	-.288	
10	↓	212.40	-.094	
11	↓	208.20	.292	
12	↓	204.20	-.541	

13 The 1D and 2D NMR spectra of 3

Figure S25 ^{1}H NMR spectrum for **3** in $CDCl_3$

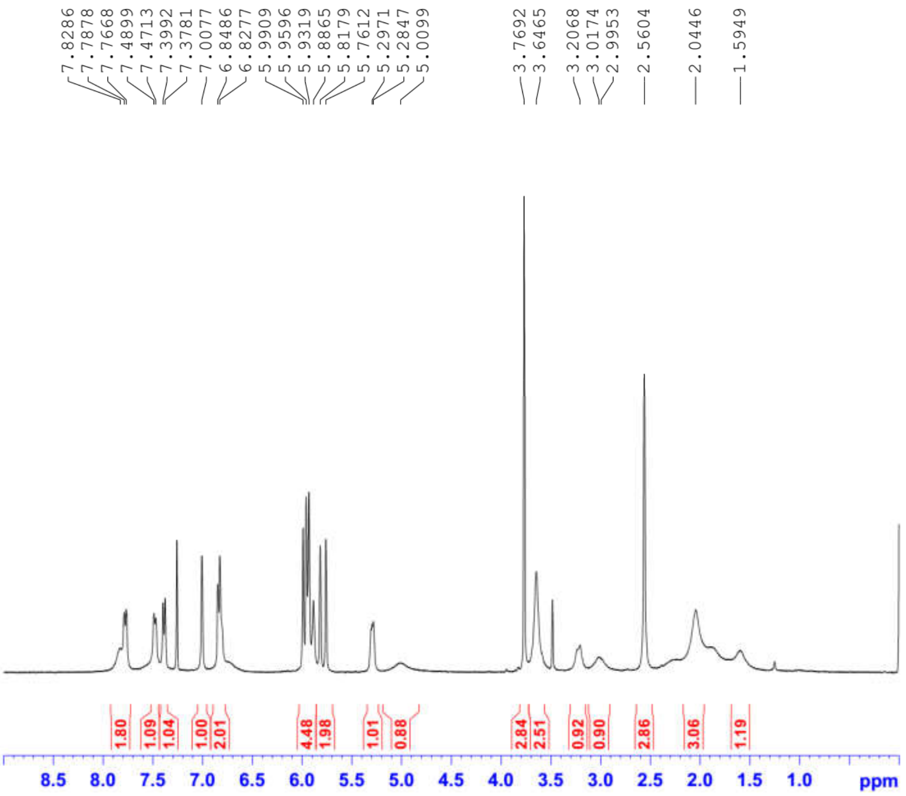

Figure S26 ^{1}H NMR spectrum for **3** in DMSO-d_6

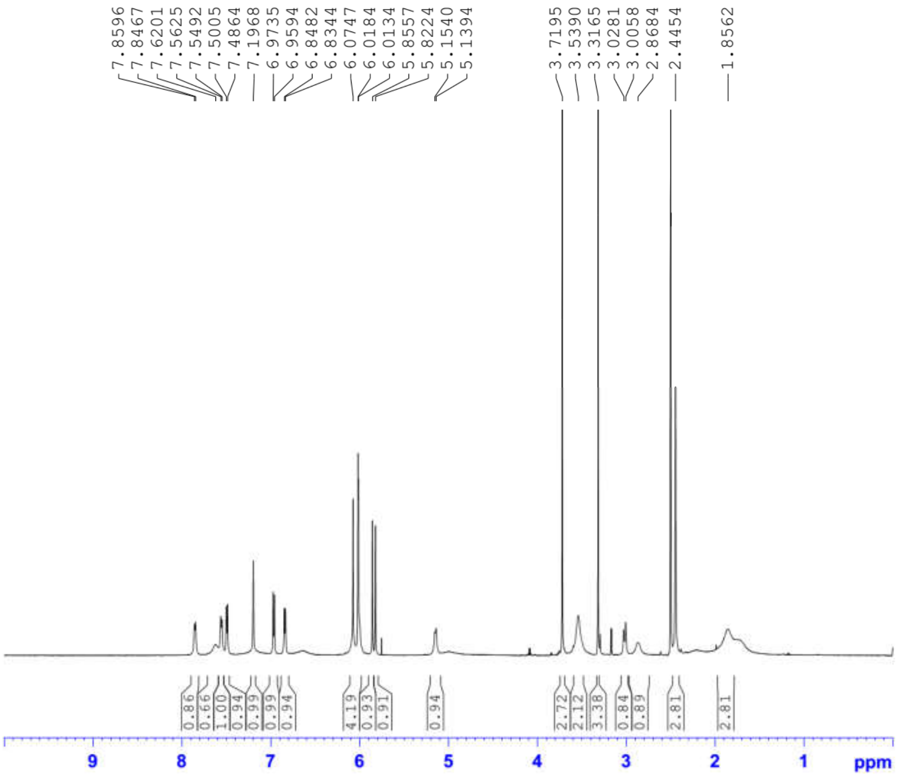

Figure S27 ^{13}C NMR spectrum for **3** in $CDCl_3$

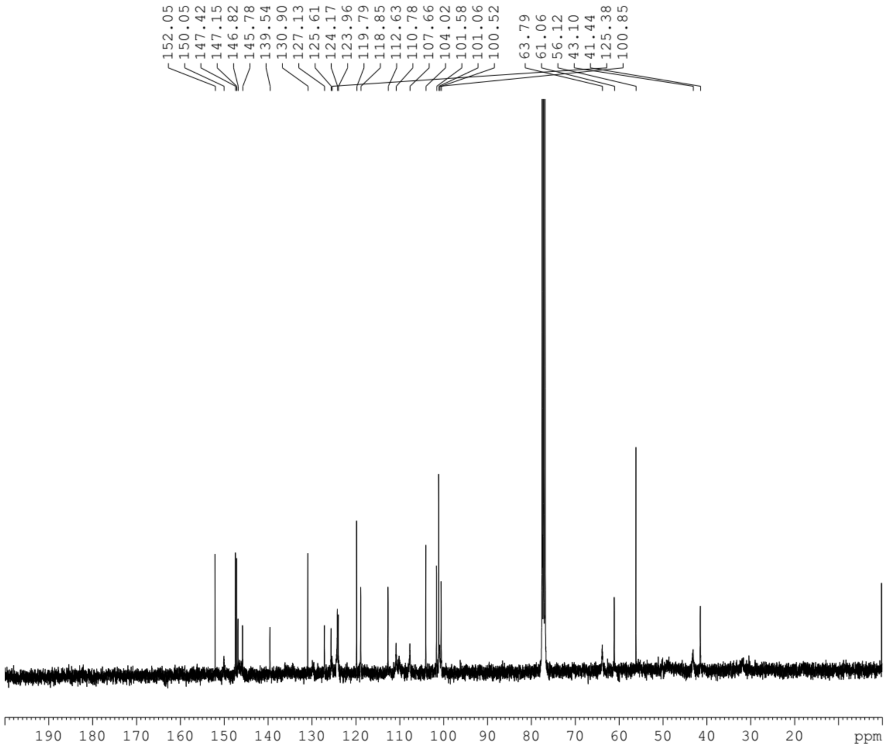

Figure S28 ^{13}C NMR spectrum for **3** in DMSO-d_6

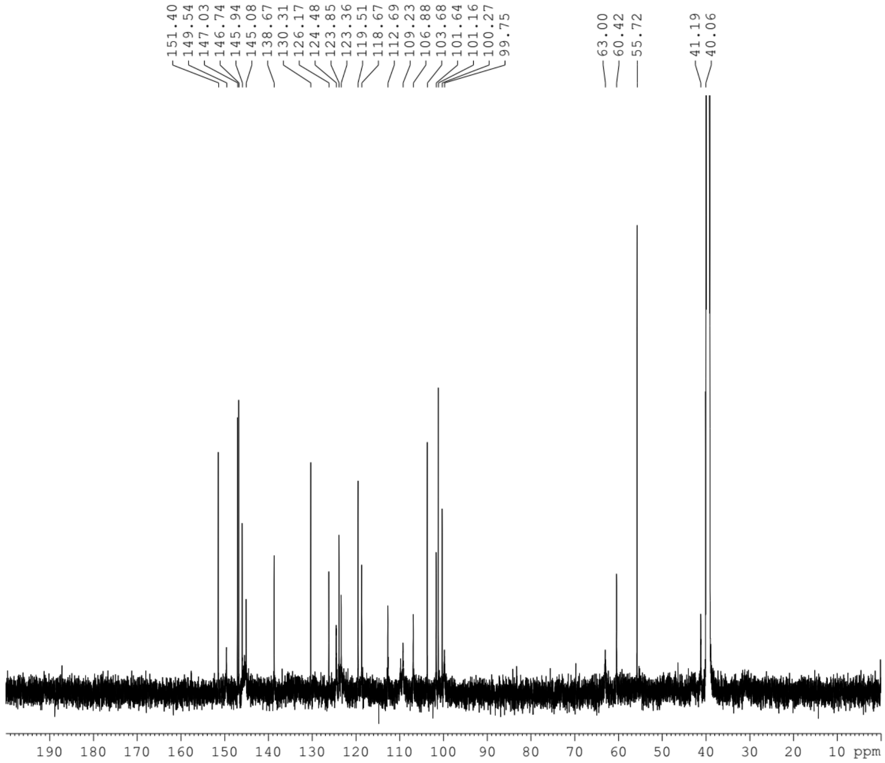

Figure S29 HSQC spectrum for **3** in DMSO-d_6

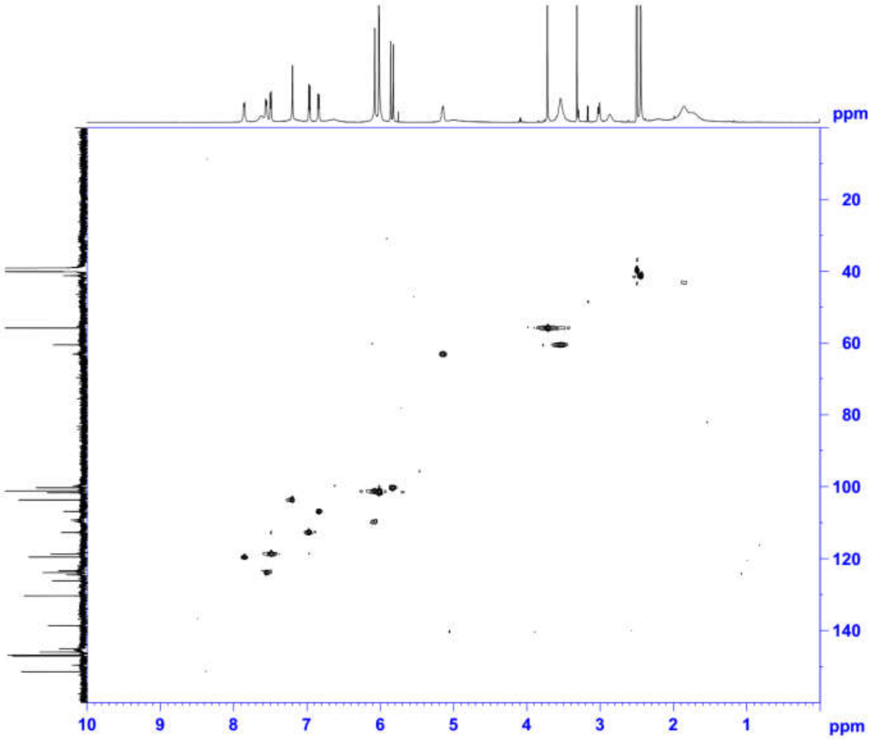

Figure S30 HMBC spectrum for **3** in DMSO-d_6

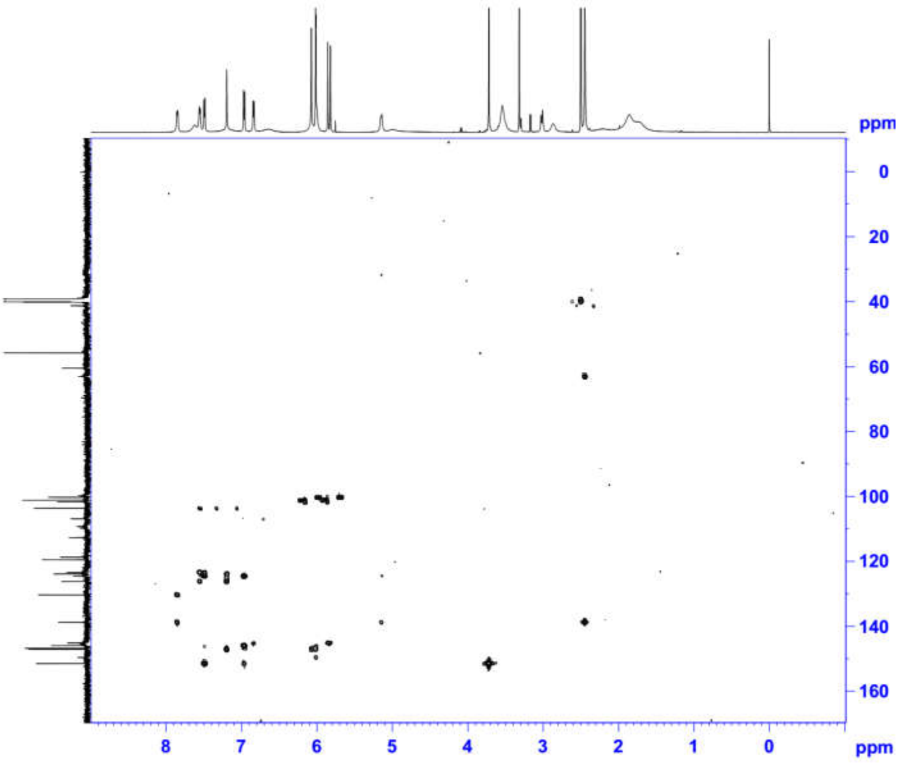

Figure S31 ^{1}H-^{1}H COSY spectrum for **3** in DMSO-d_6

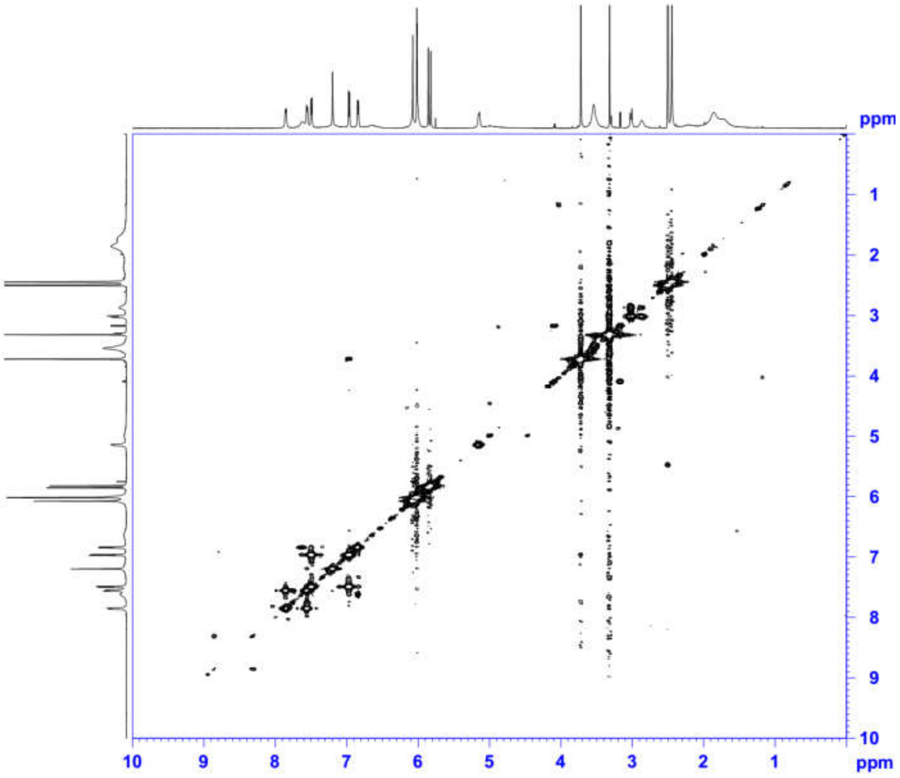

Figure S32 NOESY spectrum for **3** in DMSO-d_6

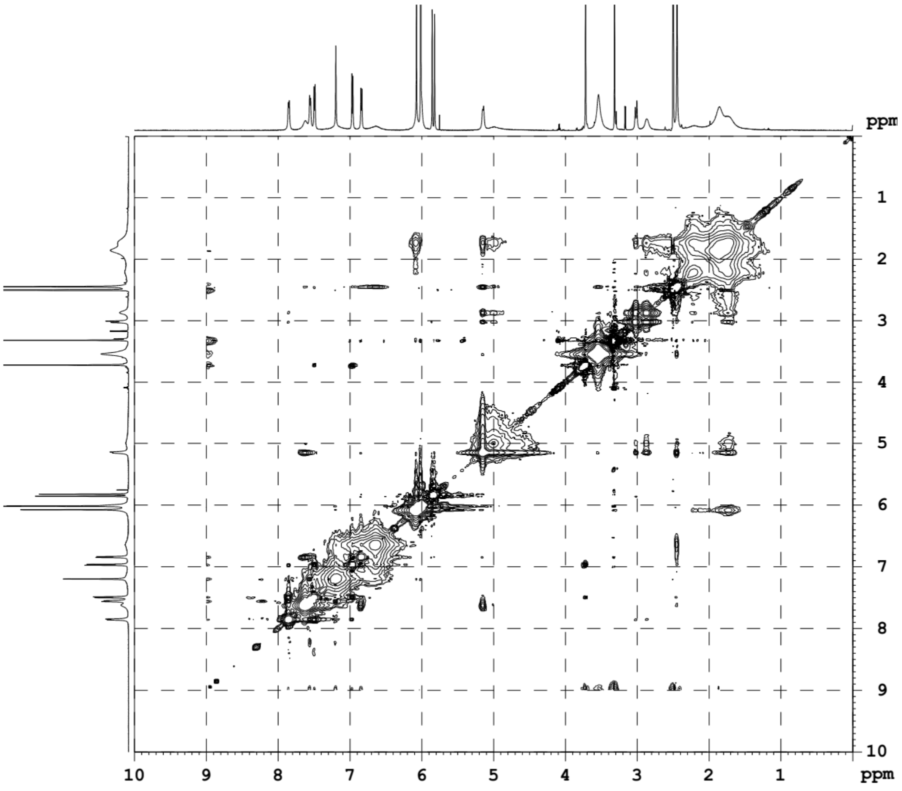

14 Figure S33 HRESIMS and HRESIMS/MS of 3

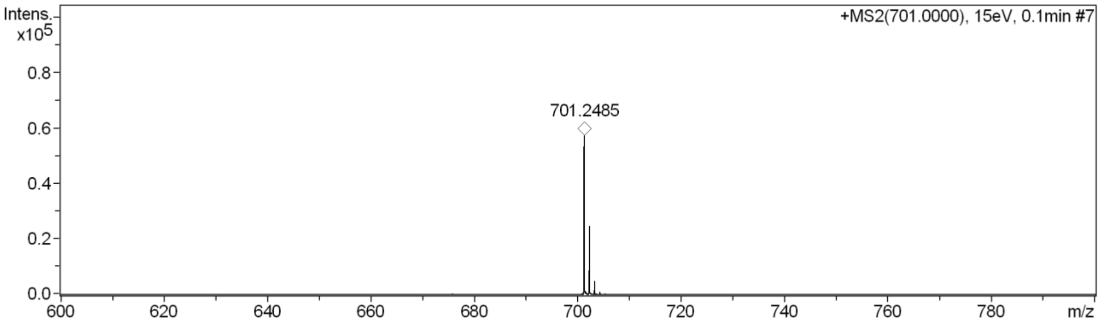

Sum Formula	Sigma	m/z	Err [ppm]	Mean Err [ppm]	Err [mDa]	rdb	N Rule	e⁻
C 41 H 37 N 2 O 9	0.026	701.2494	1.16	2.04	0.82	24.50	ok	even

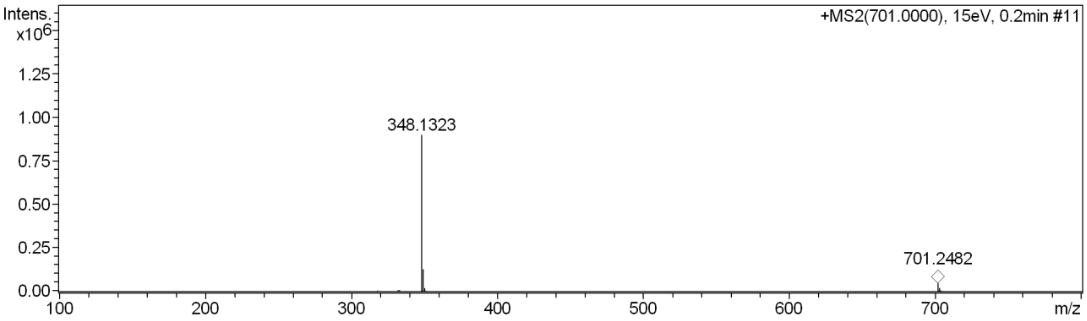

#	m/z	I
1	304.0976	1312
2	318.0764	1468
3	332.0888	5728
4	333.0981	7024
5	334.1024	1072
6	348.0215	1496
7	348.1323	898796
8	348.2723	4956
9	349.1298	127832
10	349.2399	1788
11	350.1283	18732
12	351.1305	1608
13	701.2482	42892
14	702.2488	17524
15	703.2512	3508

15 Figure S34 IR spectrum of 3

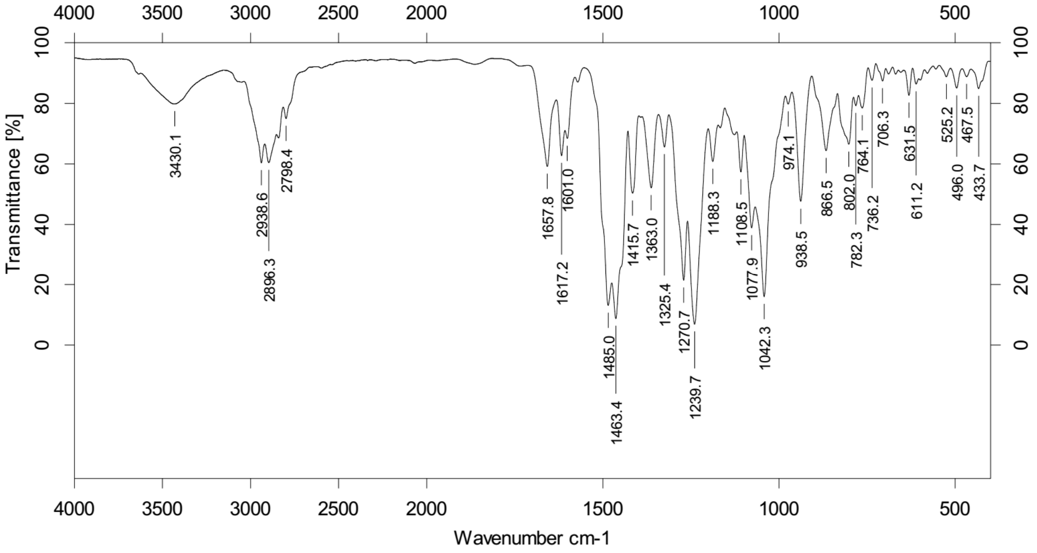

16 Figure S35 UV spectrum of 3

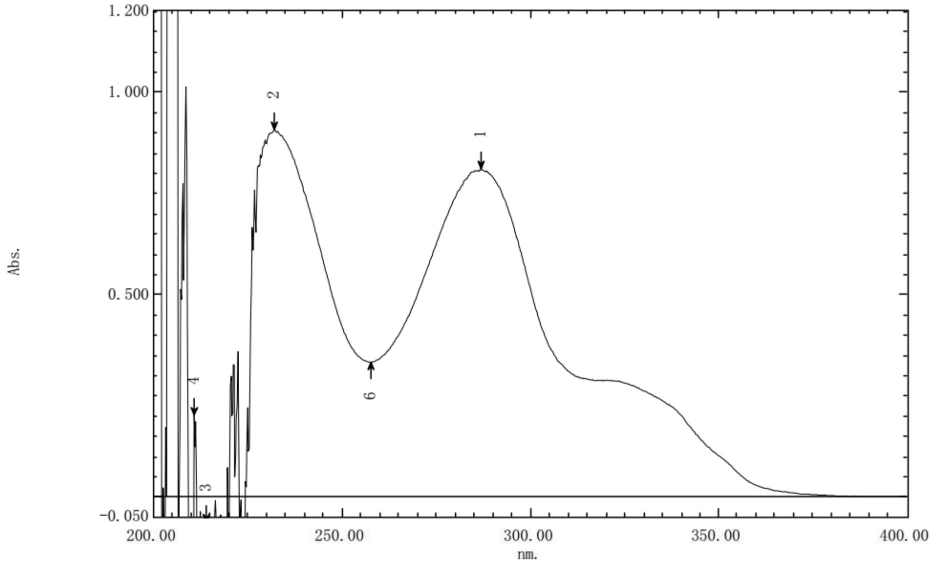

测定属性
波长范围 (nm.): 200.00到400.00
扫描速度: 中速
采样间隔: 0.2
自动采样间隔: 启用
扫描模式: 单一的

试样准备属性
重量: 0.3
体积 : 10
稀释:
光程长: 407
附加信息:

仪器属性
仪器类型: UV-1700
测定方式: 吸收值
狭缝宽: 1.0 nm
光源改变波长: 340.8 nm
S/R 转换: 标准

附件属性
附件: 无

No.	P/V	Wavelength	Abs.	描述
1	↑	286.80	.807	
2	↑	232.00	.905	
3	↑	214.20	-.066	
4	↑	210.80	.200	
5	↑	203.80	4.000	
6	↓	257.80	.331	
7	↓	219.20	-.602	
8	↓	213.00	-.602	
9	↓	209.60	-.602	
10	↓	202.20	-.576	
